# Cyclic di-AMP inhibits *Listeria monocytogenes* thymineless death during infection

**DOI:** 10.1128/mbio.03351-25

**Published:** 2025-12-11

**Authors:** Joshua P. Leeming, Omar M. Elkassih, Damilola T. Oyebode, Joshua J. Woodward, Qing Tang

**Affiliations:** 1 Department of Biology, University of Texas at Arlington 12329, Arlington, Texas, USA; 2 Department of Microbiology, University of Washington 7284, Seattle, Washington, USA; Rutgers-New Jersey Medical School, Newark, New Jersey, USA

**Keywords:** *Listeria monocytogenes*, thymineless death, cyclic di-AMP, listeriosis, infection, antimicrobial resistance, foodborne illness

## Abstract

Antifolate antibiotics are used to treat meningitis and refractory listeriosis caused by drug-resistant *Listeria monocytogenes* (*Lm*). Their bactericidal activity is attributed to the deactivation of thymidylate synthase (ThyA), which subsequently induces bacterial cell death when thymidine is depleted, a process known as thymineless death (TLD). Despite decades of study, the mechanisms of TLD, especially during infection, remain unclear. Cyclic di-AMP (c-di-AMP), a common bacterial second messenger that regulates bacterial stress responses, is elevated in response to antifolate antibiotics. In this study, we found that elevated c-di-AMP is required to inhibit TLD in *Lm*. Conversely, reducing c-di-AMP levels in the Δ*thyA* mutant led to increased bacterial cell death under thymidine starvation and significant reduction in intracellular growth. Furthermore, we found that Δ*thyA* exhibited a more pronounced growth defect during oral infection compared to intravenous infection, due to limited thymidine availability in the gallbladder, which acts as a bottleneck for Δ*thyA* in establishing infection. Notably, decreasing c-di-AMP levels abolished the infection capacity of Δ*thyA* in both infection models. Finally, we identified that the c-di-AMP-binding protein PstA contributes to bacterial cell death when c-di-AMP concentrations are low. Deletion of *pstA* in the Δ*thyA* background rescued the elevated cell death caused by c-di-AMP depletion both *in vitro* and during mouse infections. Our study identifies a previously unrecognized mechanism of TLD regulation mediated by c-di-AMP. This expands fundamental knowledge of TLD in the context of infection and provides insights into potential combined therapeutic strategies for listeriosis targeting both antifolate and c-di-AMP metabolic pathways.

**IMPORTANCE:**

Consuming food contaminated with *Listeria monocytogenes* (*Lm*) can cause severe listeriosis, a leading foodborne illness with a 20% fatality rate. Most cases require hospitalization, and 25% of pregnancy-associated cases result in fetal or neonatal death. Antibiotics, especially β-lactams, are the main treatment, but alternatives like antifolates are used when resistance or allergies occur. Still, over 30% of patients experience treatment failure, the causes of which remain poorly understood due to limited knowledge of antibiotic action within *Lm*’s intracellular niches and how the pathogen adapts during infection. This gap hinders the development of effective therapies. Our study bridges this gap by using a thymidine auxotroph mutant of *Lm* (Δ*thyA*) to investigate thymineless death both *in vitro* and *in vivo*. Notably, antifolate-resistant *Lm* strains, many of which are thymidine auxotrophs, are often found in food, posing a public health risk. Our study on how Δ*thyA* strains survive will provide insights into novel therapeutic targets.

## INTRODUCTION

*Listeria monocytogenes* (*Lm*) is a gram-positive, facultative intracellular pathogen found in natural ecosystems and widely distributed across various environmental niches (1, 2). Consuming food contaminated with *Lm* can lead to severe invasive infections, resulting in a life-threatening disease known as listeriosis (3). Listeriosis commonly presents as bacteremia, meningitis, or meningoencephalitis and includes pregnancy-associated infections, which may lead to miscarriage or neonatal sepsis (4). Listeriosis is a leading cause of death from foodborne illness, with a 20% fatality rate. Nearly all cases require hospitalization, and 25% of pregnancy-related cases result in fetal or neonatal death (5). Antibiotics remain the primary treatment for listeriosis. However, over 30% of patients experience antibiotic therapy failure (6). The underlying reasons for this failure remain unclear, particularly due to the limited understanding of how antibiotics act on the intracellular niches of *Lm* and how the bacterium adapts its physiology in response. This knowledge gap significantly hinders progress in developing optimized therapies for listeriosis.

Antifolate antibiotics are widely used in clinical practice to treat various bacterial infections. The antifolate antibiotic combination trimethoprim-sulfamethoxazole (SXT), also known as co-trimoxazole, has bactericidal activity against both extracellular and intracellular *Lm* and exhibits excellent penetration into various organs, including the central nervous system. As a result, they are used to treat meningitis and refractory listeriosis caused by drug-resistant *Lm* (7–10). Moreover, antifolate antibiotics are used for individuals who are allergic to penicillins (6, 11). Antifolate antibiotics function by inducing thymineless death (TLD) of bacteria, a phenomenon in which cells rapidly lose viability due to thymidine starvation. Despite their long-standing clinical use against listeriosis, the mechanism by which antifolates inhibit the growth of *Lm* and how bacteria respond to TLD remain largely unexplored.

TLD was first discovered in *Escherichia coli* over 70 years ago (12), and since then, *E. coli* has become the most widely used model for studying this phenomenon in prokaryotes. Although the exact mechanism of TLD remains unclear, increasing evidence suggests that it involves multiple pathways, including uncoordinated chromosomal replication initiation, central metabolism or protein synthesis (13, 14), DNA damage-response (15), reactive oxygen species (ROS) production (16), intracellular acidification (17), and abnormal cytoplasm and cell envelope (18). Despite these advances, there remain outstanding questions about the mechanisms underlying TLD in the context of infection. Notably, it is still unclear whether, and how, bacteria acquire thymidine from the host environment. *Lm* has emerged as a promising model for studying TLD during infection due to its sensitivity to antifolate antibiotics and its capacity for intracellular replication.

In response to external antibiotic stresses, *Lm* utilizes various mechanisms to rapidly and precisely alter its bacterial physiology, facilitating its survival. One such mechanism is the global regulation of bacterial physiology by the second messenger cyclic di-AMP (c-di-AMP). c-di-AMP is synthesized from two ATP molecules by diadenylate cyclase (DacA), and it can be degraded to pApA, or two AMP molecules, by specific phosphodiesterases GdpP or PdeA, respectively (19, 20). Additionally, intracellular c-di-AMP concentrations of *Lm* are regulated by multidrug transporters (MdrMTAC) that efflux this nucleotide (20). Through binding to RNA or protein regulators, c-di-AMP regulates cell wall integrity (21), osmotic homeostasis (22), and central metabolism (23). In *Lm*, both β-lactam and antifolate antibiotics have been shown to enhance c-di-AMP production (24), indicating the importance of c-di-AMP in regulating bacterial fitness against these antibiotics. This is further supported by multiple studies demonstrating that c-di-AMP level positively correlates with resistance to β-lactam antibiotics in several human pathogens (25–33). It remains unclear whether c-di-AMP functions as a regulator of antifolate antibiotic susceptibility and the underlying mechanisms involved.

In this study, we identified that c-di-AMP is a global regulator of antibiotic susceptibility to β-lactams, antifolates, and multiple antibiotics targeting protein synthesis. Our previous work showed that deactivation of ThyA, through either antifolate treatment or deletion of the thyA gene (Δ*thyA*), leads to elevated levels of c-di-AMP production (24). In this study, we further demonstrate that the elevated c-di-AMP is necessary to inhibit TLD of *Lm* Δ*thyA*. Furthermore, we found that eukaryotic host cells do not provide a sufficient thymidine source to support the intracellular growth of Δ*thyA*, which ultimately leads to a growth defect during systemic infection. We also identified that the conserved c-di-AMP-binding protein, PstA, contributes to cell death of Δ*thyA* under low c-di-AMP conditions. By investigating the role of c-di-AMP in regulating TLD during infection, our study reveals a previously unrecognized mechanism of bacterial TLD and expands fundamental knowledge of c-di-AMP signaling in cellular stress responses, offering potential therapeutic insights targeting this pathway.

## RESULTS

### c-di-AMP regulates antifolate susceptibility of *Lm*

**Fig 1:**
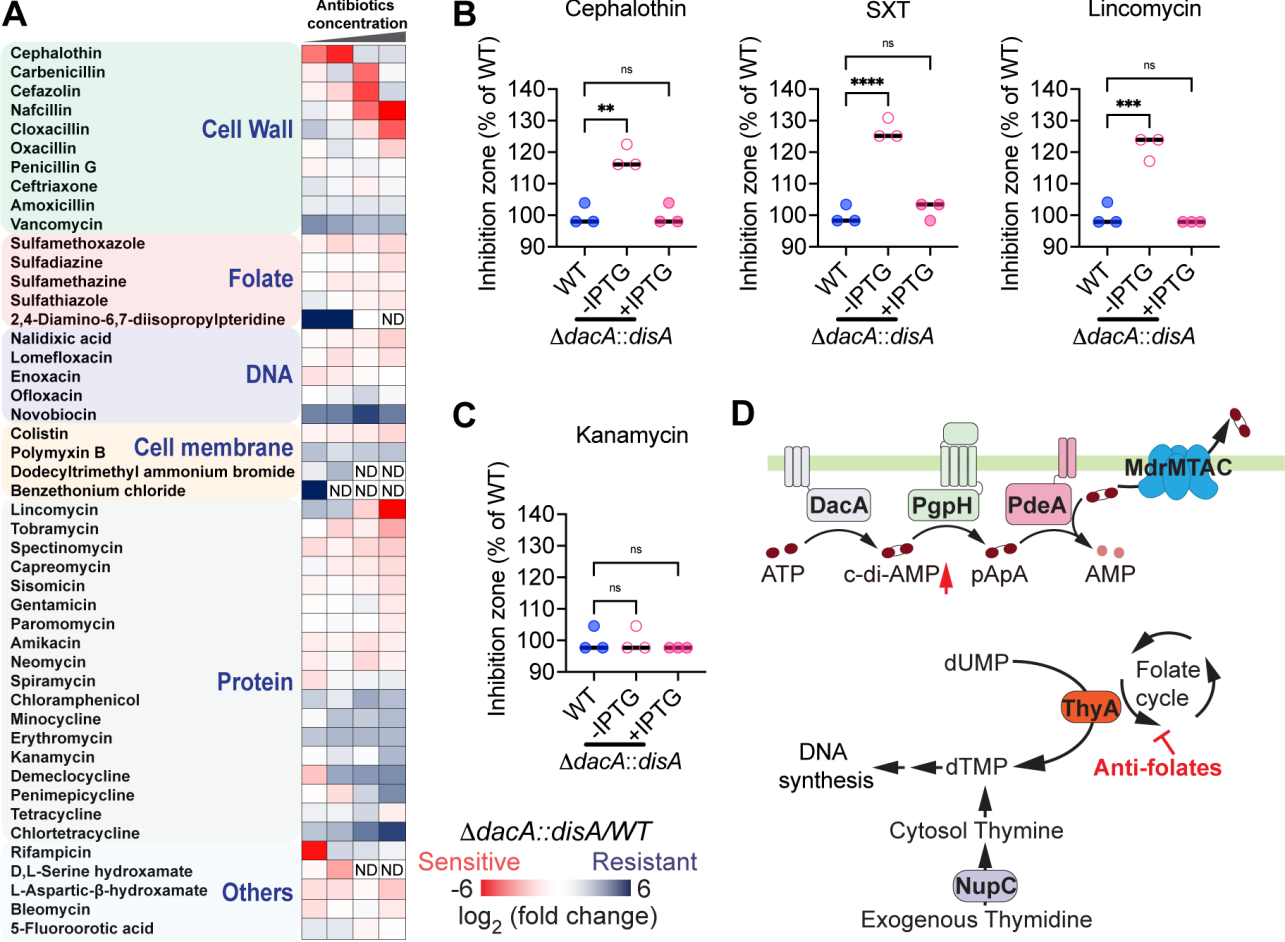
c-di-AMP functions as a global regulator for antibiotic resistance. (**A**) The heatmap indicates the log_2_ ratio of the CFU recovery of Δ*dacA::disA* (low c-di-AMP strain) relative to WT strain exposed to various antibiotics. The Δ*dacA::disA* strain, in which an IPTG-inducible DAC (DisA) replaced the original DacA, was cultured without IPTG for 16 h to halt c-di-AMP production before antibiotic treatment. The blue text indicates antibiotic classes according to their mechanisms of action, "ND" indicates that no bacteria recovery after treatment and antibiotic susceptibility was not determined. (**B**) and (**C**) antibiotic susceptibility of *Lm* strains measured by disk diffusion assays. The inhibition zone of each strain was normalized to that of the WT. For all panels, mean values of biological triplicates are plotted, and error bars indicate ±SD. *P* values were calculated using ordinary one-way analysis. Asterisks indicate that differences are statistically significant (**, *P* < 0.01; ***, *P* < 0.001; ****, *P* < 0.0001), and “ns” indicates no significant difference. (**D**) Schematic of thymidine and c-di-AMP metabolism in bacteria. ThyA, thymidylate synthase; NupC, nucleoside permease; DacA, diadenylate cyclase; PgpH, phosphodiesterase; PdeA, phosphodiesterase; MdrMTAC, multidrug transporters.

To investigate whether c-di-AMP regulates antibiotic susceptibility of *Lm*, the Δ*dacA::disA* strain, where DacA is replaced by an IPTG-inducible DisA (*Bacillus subtilis* DAC) (34), was cultured without IPTG to halt c-di-AMP production. c-di-AMP regulates various processes including osmolyte transport and bacterial cell wall integrity. These regulations subsequently affect bacterial susceptibility to β-lactam antibiotics in *S. aureus* (25, 26, 28, 29), *Lm* (21, 32), *Streptococcus pyogenes* (27, 31), and *Enterococcus faecalis* (35). Consistent with these studies, the Δ*dacA::disA* strain displayed increased susceptibility to β-lactam antibiotics, including cloxacillin, oxacillin, and penicillin G, compared to the wild-type (WT) strain. Δ*dacA::disA* was more sensitive to cephalothin, carbenicillin, and cefazolin at lower concentrations, whereas higher concentrations of these antibiotics caused dramatic inhibition in both the WT and Δ*dacA::disA* strains. Furthermore, the low c-di-AMP strain is susceptible to various categories of protein synthesis-inhibiting antibiotics, indicating the involvement of complex mechanisms. It also exhibited heightened susceptibility to most of the tested antifolate antibiotics (Fig. 1A; Table S1). This phenotype was further validated through disk diffusion assays, where treatment with β-lactam antibiotic cephalothin, antifolate antibiotic SXT, and protein synthesis-inhibitor lincomycin resulted in a larger inhibition zone on plates containing the Δ*dacA::disA* strain without IPTG induction, compared to both the Δ*dacA::disA* strain supplemented with IPTG and the WT strains (Fig. 1B). In contrast, all strains exhibited similar susceptibility to the control aminoglycoside antibiotic kanamycin (Fig. 1C). Furthermore, we observed that the Δ*dacA::disA* grown without IPTG was more sensitive to cephalothin, SXT, and lincomycin, but not to kanamycin, compared with WT strain and Δ*dacA::disA* strain grown with IPTG in brain heart infusion (BHI) broth (Fig. S1).

Antifolates disrupt the synthesis of 5,10-methylenetetrahydrofolate, an essential co-factor of ThyA. ThyA synthesizes the DNA synthesis precursor dTMP from dUMP. Disruption of the folate cycle inhibits the enzymatic activity of ThyA, resulting in reliance on exogenous thymidine to maintain DNA synthesis (Fig. 1D). When deprived of thymidine, ThyA defective strains lose viability, known as TLD. Inactivating ThyA by antifolate inhibition or *thyA* gene deletion leads to elevated c-di-AMP production in *Lm* (24) (Fig. 1D)*,* suggesting that *Lm* may increase c-di-AMP production under thymidine deprivation to inhibit TLD.

### c-di-AMP is crucial for maintaining cell viability during thymidine deprivation *in vitro*

**Fig 2:**
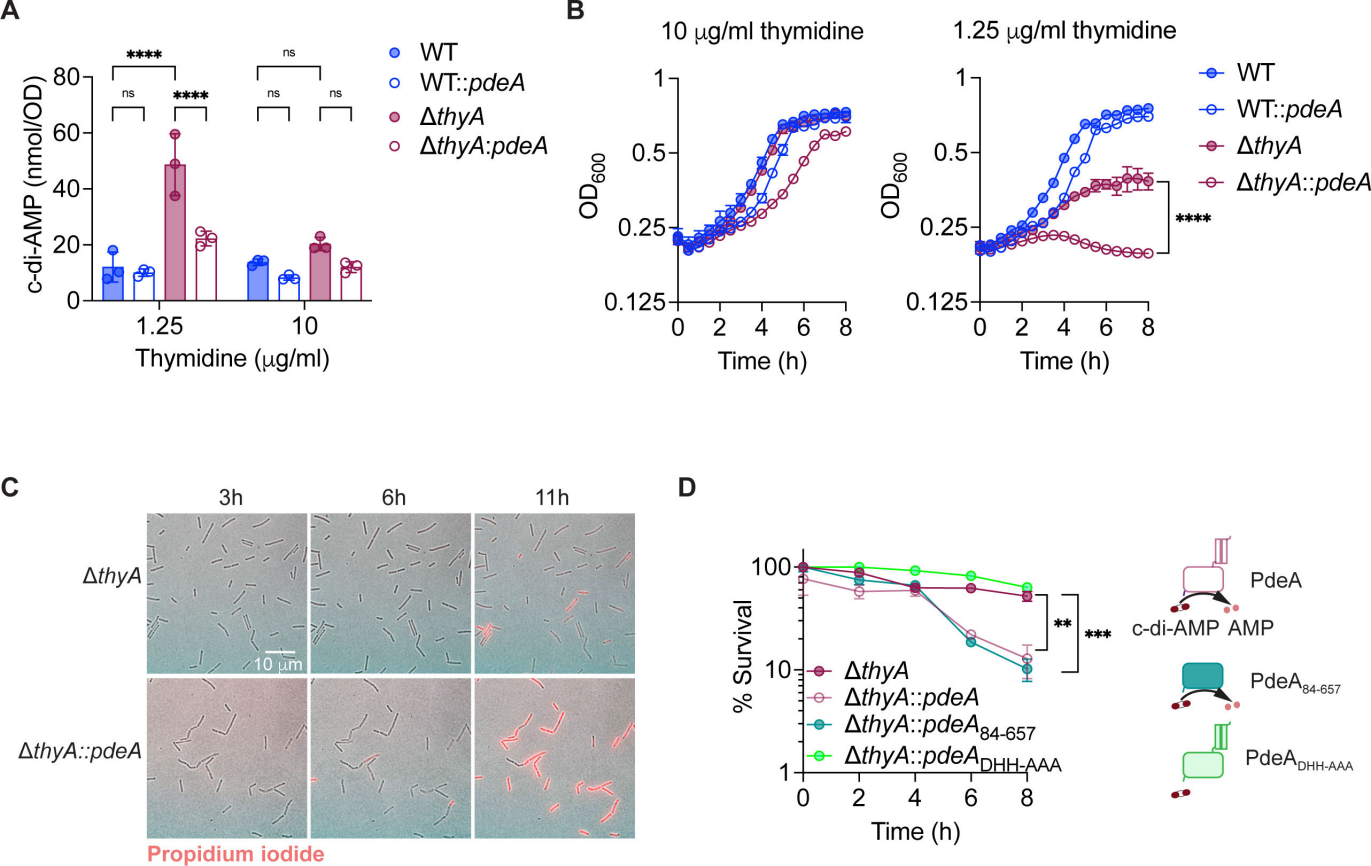
c-di-AMP is crucial for maintaining cell viability during thymidine deprivation *in vitro*. (**A**) Intracellular c-di-AMP concentration of *Lm* strains quantified using LC-MS. The *Lm* strains were grown overnight statically at 30 °C in BHI broth supplemented with different concentrations of thymidine. (**B**) Growth curves of *Lm* strains with varying concentrations of thymidine supplementation in BHI broth, incubated statically at 30 °C. (**C**) Composite phase-contrast and red fluorescence images of *Lm* strains stained with propidium iodide. Δ*thyA* and Δ*thyA::pdeA* strains were grown on lysogeny broth (LB) agar without thymidine and supplemented with propidium iodide during the 11 hours of imaging, which penetrates only the cell envelopes of dead cells. (**D**) Relative CFU recovery of bacteria incubated in LB without thymidine. The percentage of survival at each time point was calculated by normalizing the CFU count to the value at time 0. PdeA, c-di-AMP phosphodiesterase, PdeA_84–657_, enzymatic domain of PdeA. PdeA_DHH-AAA_, enzymatic dead mutant of PdeA. For all panels, mean values of biological triplicates are plotted, and error bars indicate ±SD. *P* values were calculated using either two-way ANOVA (**A**) or ordinary one-way ANOVA (**B and D**). Asterisks indicate that differences are statistically significant (**, *P* < 0.01; ***, *P* < 0.001, ****, *P* < 0.0001), and “ns” indicates no significant difference.

Given that inactivation of ThyA by deleting its encoding gene is equivalent to inhibiting ThyA with antifolates, we generated an *Lm thyA* knockout strain (Δ*thyA*) to study the mechanism of TLD. *Lm* Δ*thyA* produces significantly higher levels of c-di-AMP compared to the WT strain (24). c-di-AMP depletion is detrimental to the growth of *Lm* (32). As a result, we generated WT::*pdeA* and Δ*thyA::pdeA* strains, in which we overexpressed the phosphodiesterase PdeA to reduce, but not completely eliminate, c-di-AMP production (Fig. 2A; Fig. S2). We found that the Δ*thyA::pdeA* strain exhibited only a moderate growth delay compared to the Δ*thyA* strain when supplemented with a high concentration of exogenous thymidine (10 µg/mL) (Fig. 2B, left); however, the growth defect was exacerbated under lower thymidine concentration (1.25 µg/mL) (Fig. 2B, right). In contrast, in the WT strain with functional ThyA, decreasing c-di-AMP did not affect bacterial growth regardless of thymidine supplementation (Fig. 2B). Additionally, we found that Δ*thyA::pdeA* exhibited significantly higher membrane integrity loss compared to the Δ*thyA* strain after 11 hours of thymidine starvation, as assessed by propidium iodide staining (Fig. 2C and Videos S1 and S2). To determine whether the membrane integrity loss was due to cell death, we monitored the colony-forming unit (CFU) recovery in Δ*thyA*-derived strains following thymidine starvation. Reducing c-di-AMP levels by overexpressing either the full-length (Δ*thyA::pdeA*) or the enzymatic domain of its phosphodiesterase in the Δ*thyA* strain (Δ*thyA::pdeA*_84–657_) accelerated cell death. Conversely, overexpression of the enzymatically inactive PdeA mutant (Δ*thyA::pdeA*_DHH-AAA_) had no effect on cell viability (Fig. 2D). These data suggest that elevated c-di-AMP production in Δ*thyA* is crucial for maintaining cell viability when cells are deprived of thymidine and that decreasing c-di-AMP to the normal level leads to cell death.

### c-di-AMP is required for the intracellular growth of Δ*thyA* in tissue cultures

*Lm* is a highly adaptable bacterium that replicates as a facultative intracellular pathogen. Its intracellular replication within the host is essential for its pathogenesis. However, it remains unclear whether *Lm* can acquire sufficient thymidine from host cells to support its intracellular growth in response to antifolate antibiotics.

**Fig 3:**
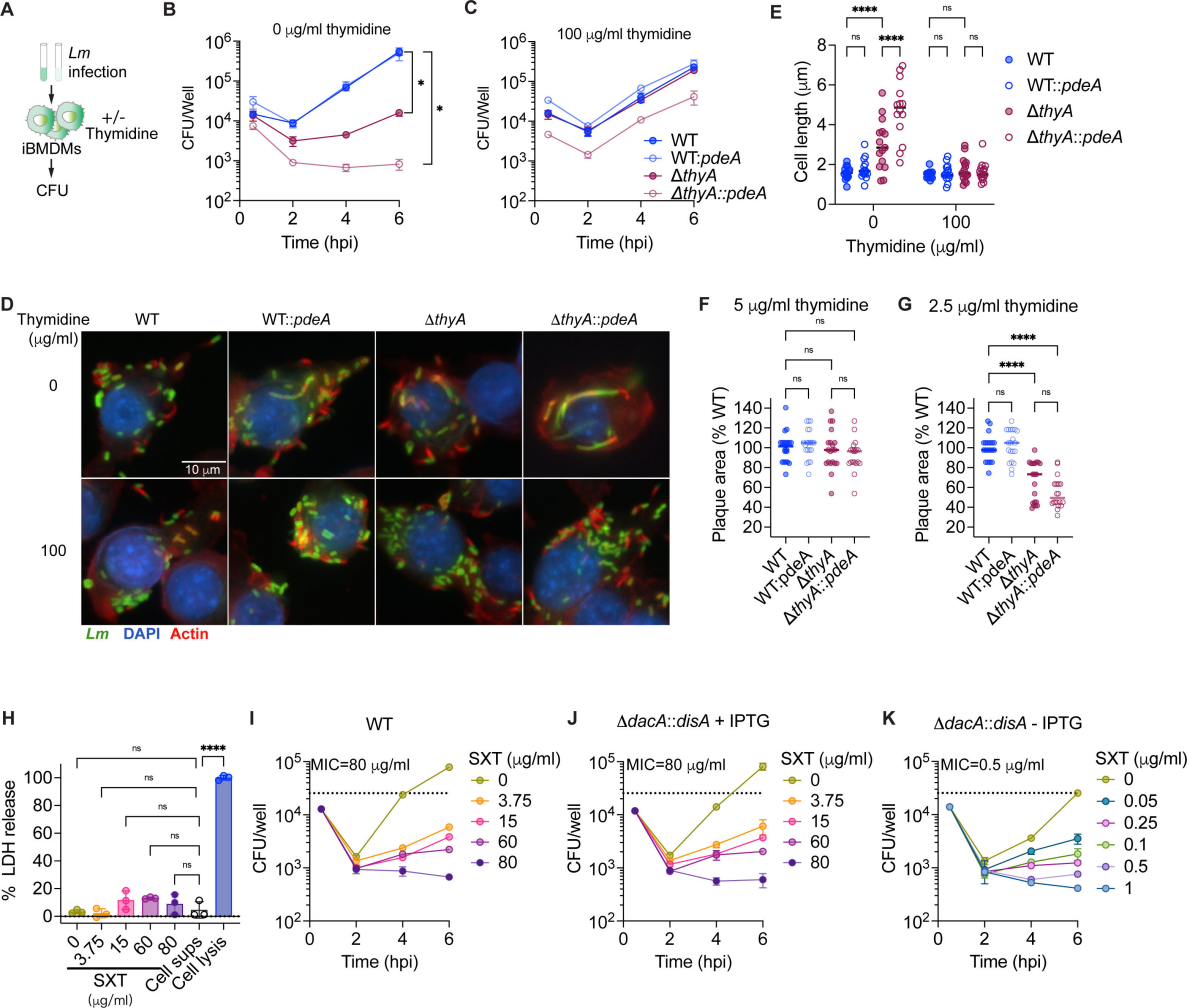
Elevated c-di-AMP production is required for the intracellular growth of Δ*thyA*. (**A**) Schematic of macrophage infection with *Lm*. The iBMDMs were infected with *Lm* at a multiplicity of infection (MOI) of 1 in the presence or absence of extracellular thymidine supplementation. Gentamicin was supplemented at 0.5 hours post-infection (hpi) to kill the extracellular bacteria. (**B**) and (**C**) Intracellular growth curve of *Lm* in iBMDMs, as described in **A**. Mean values of biological duplicates are plotted. (**D**) Fluorescence microscopy images of iBMDMs infected with *Lm* at an MOI of 10 at 6 hpi. Nuclei were stained with DAPI (blue), F-actin with Alexa Fluor 568 phalloidin (red), and *Lm* with anti-*Lm* antibody (green). (**E**) Cell length quantification of intracellular *Lm,* as indicated in **D**. Mean values of cell length of 15 bacterial cells from three different macrophages were plotted. (**F**) and (**G**) Plaque size of L2 fibroblasts with *Lm* infection. The monolayer of fibroblasts was infected with *Lm* for 2 days with gentamicin and various concentrations of thymidine supplementation and then stained with neutral red. The plots represent the median of 20 plaques measured from three different wells, with plaque diameters normalized to the control. (**H**) Lactate dehydrogenase (LDH) release from iBMDMs treated with increasing concentrations of SXT (in DMSO) normalized to cell lysis controls. “Cell sups” indicates the cell supernatant from untreated macrophages. Mean values of biological triplicates are plotted. (**I**), (**J**), and (**K**) Intracellular growth curve of *Lm* in iBMDMs with or without SXT treatment. The iBMDMs were infected with *Lm* with an MOI of 1 in the presence or absence of IPTG supplementation. Gentamicin and SXT were supplemented at 0.5 hpi. Mean values of biological duplicates are plotted. For all panels, error bars indicate ±SD*, P* values were calculated using ordinary one-way ANOVA (**B, C, F, G, H**) or two-way ANOVA (**E**). Asterisks indicate that differences are statistically significant (*, *P* < 0.05; **, *P* < 0.01; ****, *P* < 0.0001), and “ns” indicates no significant difference.

To investigate whether c-di-AMP is essential for the intracellular growth of *Lm*, immortalized bone marrow-derived macrophages (iBMDMs) were infected with WT, WT::*pdeA*, Δ*thyA*, and Δ*thyA::pdeA* strains in the presence or absence of extracellular thymidine (Fig. 3A). In the absence of thymidine, Δ*thyA* showed a significant growth defect compared to the WT strain. Notably, Δ*thyA::pdeA* totally lost its ability to replicate in iBMDMs (Fig. 3B). Nevertheless, supplementation with extracellular thymidine restored the growth defect of both strains (Fig. 3C). Additionally, PdeA overexpression in the WT strain did not affect its intracellular growth, with both the WT and WT::*pdeA* strains showing similar growth trends regardless of thymidine supplementation (Fig. 3B and C). Moreover, after 6 h of intracellular growth in macrophages, the WT, WT::*pdeA*, and Δ*thyA* strains exhibited a similar bacterial morphology, while Δ*thyA::pdeA* cells displayed cell elongation and swelling (Fig. 3D and E). Cell elongation or swelling under thymidine starvation has been observed across multiple bacterial species (18, 36). Supplementation with exogenous thymidine fully restored the normal morphology of Δ*thyA::pdeA* cells (Fig. 3D and E). In addition, the elongated Δ*thyA::pdeA* cells were able to recruit actin tails, which facilitate intracellular movement and cell-to-cell spread (Fig. 3D). These results indicate that Δ*thyA* could not obtain enough thymidine to support its intracellular growth, while c-di-AMP is essential for inhibiting bacterial cell death caused by thymidine deprivation.

In addition to macrophages, *Lm* invades and grows in a variety of mammalian cells, including epithelial cells and fibroblasts. *Lm* spreads intracellularly and forms small plaques in monolayers of fibroblasts (37). To determine if c-di-AMP is essential for the growth and intracellular spreading of Δ*thyA*, mouse L2 fibroblasts were infected with *Lm* to measure plaque sizes. Consistent with the defective intracellular growth in macrophages, L2 cells infected with Δ*thyA* did not form any plaques in the absence of exogenous thymine (Fig. S3). However, supplementation with 5 µg/mL extracellular thymidine fully restored the plaque-forming ability of Δ*thyA* (Fig. 3F; Fig. S3). Moreover, with a lower concentration of thymidine supplementation (2.5 µg/mL), plaque sizes of both Δ*thyA* and Δ*thyA::pdeA* are significantly smaller than those formed by the WT and WT::*pdeA* strains. Δ*thyA::pdeA* exhibited a moderate, but not significant, reduction in plaque size compared to Δ*thyA* (Fig. 3G; Fig. S3). These results indicate that c-di-AMP does not impact the intracellular spreading of Δ*thyA*, as further supported by the observation that Δ*thyA::pdeA* forms actin tails (Fig. 3D).

Our data indicate that eukaryotic host cells cannot supply sufficient thymidine to support the intracellular growth of Δ*thyA* and that c-di-AMP is required to inhibit TLD. We hypothesize that c-di-AMP is also essential for *Lm* survival during antifolate antibiotic treatment. iBMDMs infected with WT or Δ*dacA::disA* strains, with or without IPTG supplementation, were treated with different concentrations of SXT at 0.5 hpi. SXT alone does not exhibit significant cytotoxicity to the cells, as assessed by measuring LDH release in the cell supernatants (Fig. 3H). Consistent with our hypothesis, the minimum inhibitory concentration (MIC) of SXT required to inhibit intracellular growth in iBMDMs is 80 µg/mL for both WT *Lm* and the Δ*dacA::disA* strain supplemented with IPTG, whereas the MIC for the Δ*dacA::disA* strain without IPTG induction is markedly lower at 0.5 µg/mL (Fig. 3I through K). When SXT is used clinically, its mean blood levels range from 98 to 120 μg/mL during the first 8 h following a single oral dose (38), which is 196- to 240-fold higher than the MIC for Δ*dacA::disA* without IPTG. As a result, c-di-AMP synthesis is a potential drug target that could significantly enhance the efficacy of antifolates in combination therapies.

### c-di-AMP is required for the growth of Δ*thyA* during mouse infection

**Fig 4:**
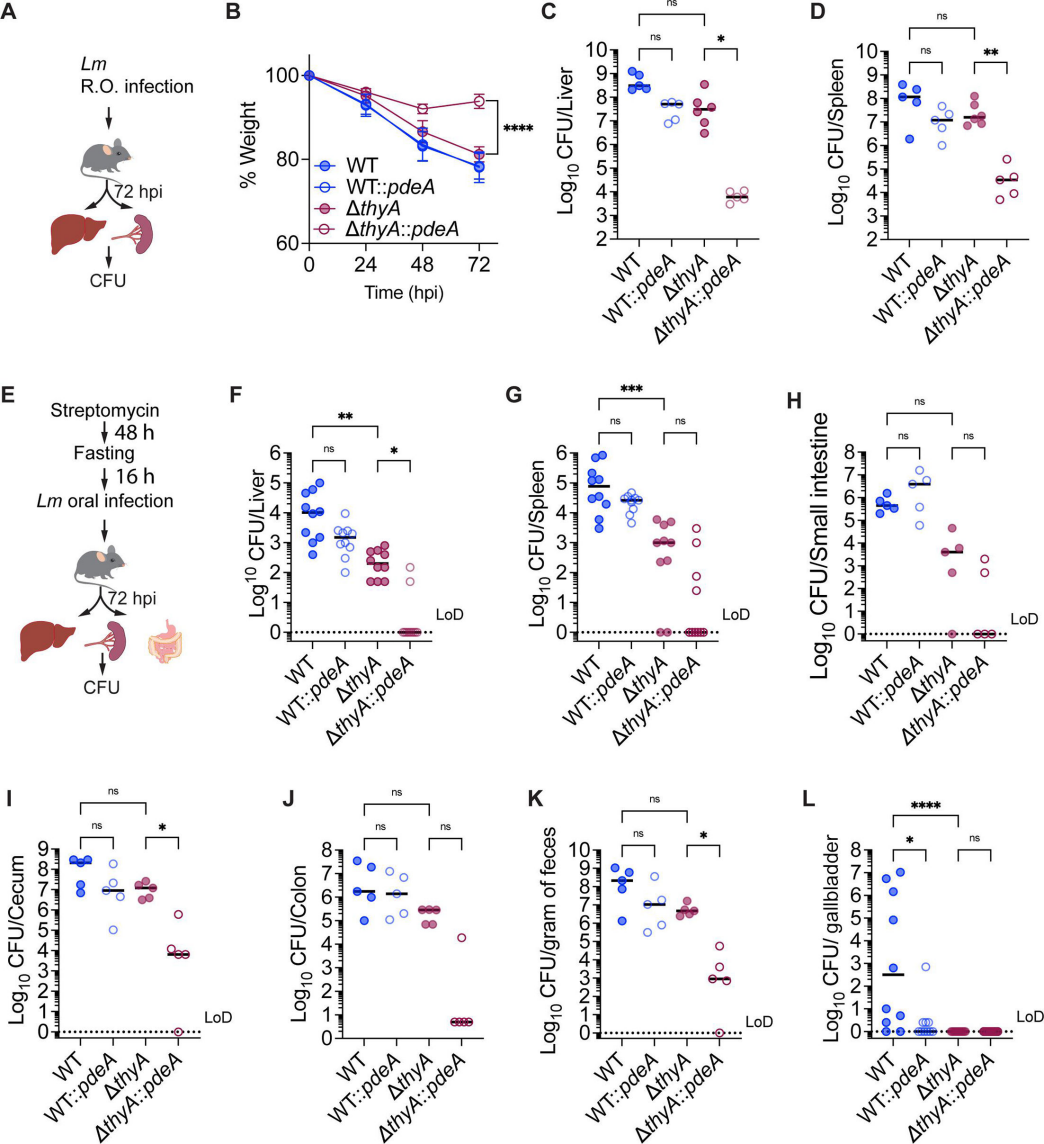
Elevated c-di-AMP production is required for the growth of Δ*thyA* during mouse infection*.* (**A**) Schematic of R.O. *Lm* infection in mice. C57BL/6 mice were infected with 10^5^ CFU/mouse via the R.O. route. Livers and spleens were harvested for CFU enumeration at 72 hpi. (**B**) Weight loss of infected mice, as indicated in panel **A**. (**C**) and (**D**) CFU recovery of *Lm* from spleens and livers, as described in **A**. (**E**) Schematic of oral *Lm* infection in mice. C57BL/6 mice were given 5 mg/mL streptomycin in drinking water for 48 h, fasted overnight, and then orally inoculated with 10^8^ *Lm*. Livers and spleens were harvested for CFU enumeration at 72 hpi. (**F**) to (**L**) CFU recovery of *Lm* from mouse tissue or feces samples, as described in **E**. For panel **B**, mean values of five biological replicates are plotted, and error bars indicate ±SD. *P* values were calculated using ordinary one-way ANOVA. For all CFU recovery in mouse infections, biological replicates are plotted. Horizontal black bars indicate the median of the data. *P* values were calculated using Kruskal-Wallis followed by uncorrected Dunn’s test. Asterisks indicate that differences are statistically significant (*, *P* < 0.05; **, *P* < 0.01; ***, *P* < 0.001, ****, *P* < 0.0001), and “ns” indicates no significant difference. “LoD” denotes the limit of detection.

To investigate whether c-di-AMP is required by Δ*thyA* to maintain bacterial growth *in vivo*, C57BL/6 mice were retro-orbitally (R.O.) infected with WT, WT::*pdeA*, Δ*thyA*, and Δ*thyA::pdeA* strains with an inoculum of 10^5^ CFU/mouse, followed by CFU enumeration of the liver and spleen at 72 hpi (Fig. 4A). All strains caused weight loss upon infection; however, the weight of Δ*thyA::pdeA*-infected mice stopped decreasing at 48 hpi (Fig. 4B), indicating the dampened ability of this strain to cause listeriosis. The Δ*thyA* strain exhibited robust growth in both the liver and spleen, indicating that it had access to thymidine *in vivo* to support its proliferation. Notably, reducing c-di-AMP levels by overexpressing PdeA in the WT strain only slightly impaired its growth but completely abolished the growth of Δ*thyA* in both the spleen and liver (Fig. 4C and D), which is consistent with the reduced weight loss observed in mice infected with Δ*thyA::pdeA* (Fig. 4B).

Intravenous *Lm* infection bypasses the intestinal epithelial barrier and directly enters the bloodstream. To determine whether c-di-AMP is required for Δ*thyA* to disseminate from the intestine to peripheral tissues, we orally infected C57BL/6 mice with the same strains with an inoculum of 10^8^ CFU/mouse and monitored the CFU recovery from mouse tissues at 72 hpi (Fig. 4E). Δ*thyA* exhibited a more pronounced growth defect during oral infection compared to R.O. infection, with approximately a 2-log decrease in CFU recovery from both the liver and spleen compared to the WT. Consistently, we observed that Δ*thyA::pdeA* showed an even more severe growth impairment compared to Δ*thyA*, with most mice displaying CFU levels in the liver and spleen below the limit of detection (Fig. 4F and G). Surprisingly, while Δ*thyA::pdeA* showed a pronounced defect in establishing infection in the gastrointestinal tract, Δ*thyA* exhibited comparable colonization in the cecum, colon, and feces relative to the WT (Fig. 4H through K). As the gallbladder is an important reservoir for *Lm* during oral infection (39), we found that neither Δ*thyA* nor Δ*thyA::pdeA* exhibited growth in the gallbladder (Fig. 4L). This suggests that the gallbladder is deficient in pyrimidines, which are necessary to support the growth of Δ*thyA* mutants. This deficiency may contribute to their more pronounced defect in dissemination to the liver and spleen during oral infection compared to R.O. infection. Furthermore, WT::*pdeA* exhibited significantly reduced survival in the gallbladder compared to the WT (Fig. 4L), indicating that c-di-AMP may also play a critical role in bile acid resistance.

Taken together, our data indicate that the gallbladder is a significant bottleneck for Δ*thyA* in establishing infection, whereas c-di-AMP is required for Δ*thyA* to colonize the intestines, disseminate from the gastrointestinal tract to peripheral organs, and grow in these organs.

### PstA promotes TLD under low c-di-AMP concentrations

Previous studies have identified the PII family signaling protein PstA as a conserved c-di-AMP binder in Firmicutes (40–42). PII signaling proteins typically regulate cellular processes through protein-protein interactions that are inhibited upon metabolite binding (43). Although the specific targets of PstA remain unknown, deletion of PstA partially suppresses *Lm* cell death under c-di-AMP deprivation in rich medium (33, 41). We hypothesize that PstA plays a role in regulating the bacterial stress response, including thymineless death, downstream of c-di-AMP signaling.

**Fig 5:**
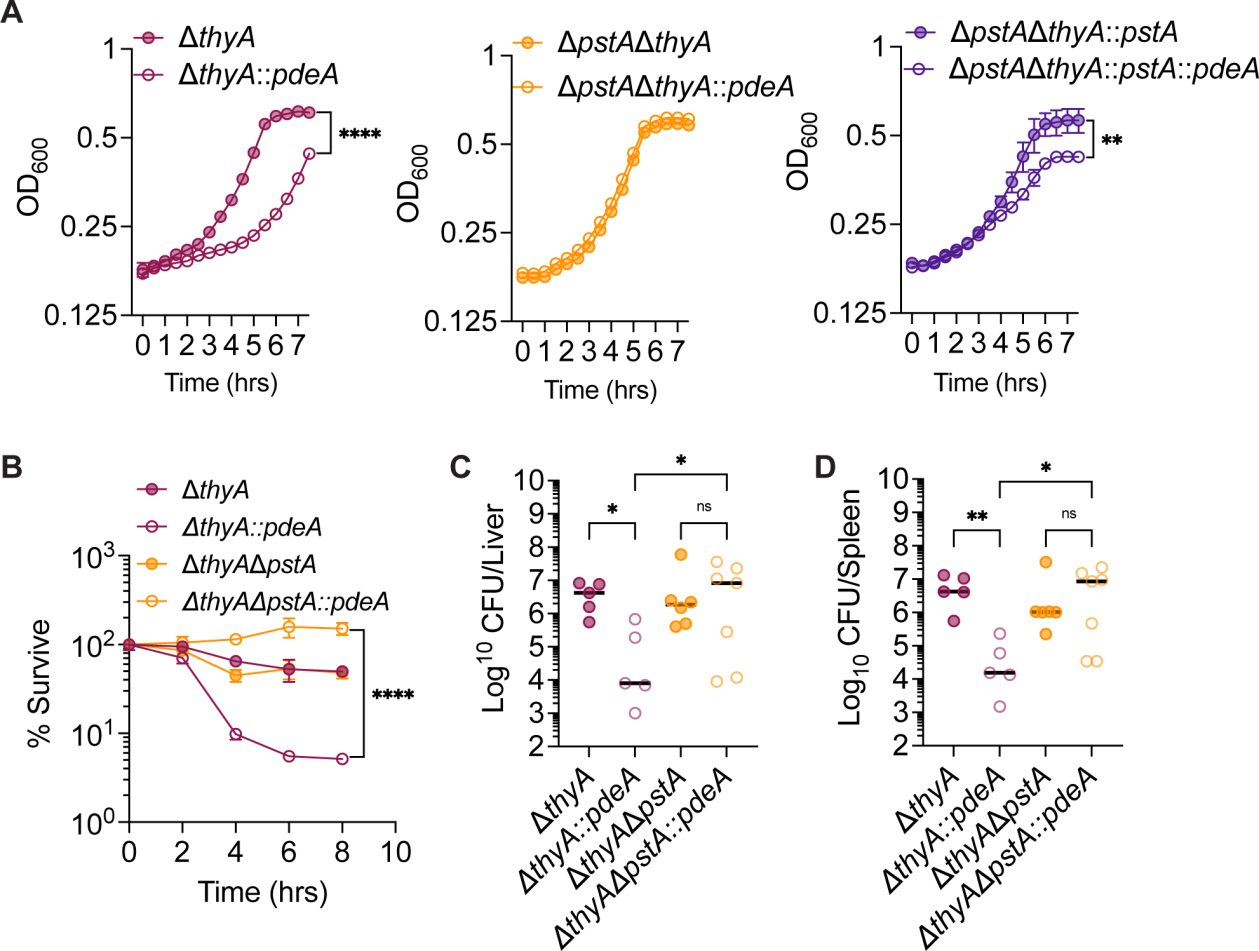
PstA promotes thymineless death in the absence of c-di-AMP. (**A**) Growth curve of *Lm* strains grown in BHI with 2.5 µg/mL thymidine. (**B**) Bacterial survival in the absence of thymidine. The survival rate at each time point was calculated by normalizing the CFU counts to the value at time 0. (**C**) and (**D**) CFU recovery of *Lm* from spleens and livers. C57BL/6 mice were infected with 10⁵ CFU *Lm* per mouse via the R.O. route. Livers and spleens were harvested for CFU enumeration at 72 hpi. For panels **A** and **B**, mean values of biological triplicates are plotted, and error bars indicate ±SD*, P* values were calculated using two-way ANOVA. For all mouse infection panels, biological replicates are plotted. Horizontal black bars indicate the median of the data. *P* values were calculated using Kruskal-Wallis followed by uncorrected Dunn’s test. Asterisks indicate that differences are statistically significant (*, *P* < 0.05; **, *P* < 0.01; ****, *P* < 0.0001), and “ns” indicates no significant difference.

We found that the Δ*thyA::pdeA* strain exhibited a growth defect compared to the Δ*thyA* strain when grown in low thymidine concentrations (2.5 µg/mL). While deletion of *pstA* in the Δ*thyA* background had no effect on growth, its deletion in the Δ*thyA::pdeA* strain fully rescued the growth defect. Furthermore, complementation of *pstA* in the Δ*thyA*Δ*pstA::pdeA* strain led to a significant growth defect compared to the Δ*thyA*Δ*pstA::pdeA* strain, further supporting that the growth impairment is attributable to PstA (Fig. 5A). Similarly, deletion of *pstA* restored the survival of the Δ*thyA::pdeA* strain in the absence of exogenous thymidine (Fig. 5B). This phenotype was also observed in the Δ*thyA*Δ*dacA::dacA* strain, in which the Δ*dacA* mutation was complemented with an IPTG-inducible *dacA*. In the absence of both thymidine and IPTG, Δ*thyA*Δ*dacA::dacA* exhibited a significant survival defect compared to the Δ*thyA* strain, while *pstA* deletion rescued this defect (Fig. S4). These data indicate that either overexpression of PdeA or deletion of DacA, both of which reduce c-di-AMP levels, led to a significant cell death of Δ*thyA* in the presence of PstA.

To investigate whether PstA affects the *in vivo* growth of Δ*thyA*, C57BL/6 mice were R.O. infected with Δ*thyA*, Δ*thyA::pdeA*, Δ*thyA*Δ*pstA*, and Δ*thyA*Δ*pstA::pdeA* strains at an inoculum of 10⁵ CFU per mouse. Consistent with the *in vitro* survival assays, deletion of *pstA* fully restored the growth defect of Δ*thyA::pdeA in vivo*, with CFU recovery in both the spleen and liver comparable to that of the Δ*thyA* strain (Fig. 5C and D).

Taken together, our data suggest that PstA is detrimental to the survival of Δ*thyA* in the absence of c-di-AMP. Elevated c-di-AMP levels in Δ*thyA* appear to counteract the toxic effect of PstA.

## DISCUSSION

The results of this study demonstrate that c-di-AMP is essential for the survival of *Lm* in response to multiple categories of antibiotics, including β-lactams, antifolates, and protein synthesis inhibitors. Our previous work showed that deactivation of ThyA, through either antifolate treatment or deletion of the *thyA* gene (Δ*thyA*), leads to elevated levels of c-di-AMP (24). In this study, we further demonstrate that the elevated c-di-AMP is necessary to inhibit the toxic activity of PstA and subsequently counteract cell death of Δ*thyA* under thymidine-deprived conditions and during intracellular growth.

TLD underlies the effectiveness of several antibacterial (trimethoprim and sulfamethoxazole), antimalarial (pyrimethamine and sulfonamide), anticancer (methotrexate and fluorouracil), and immune-modulating (methotrexate) agents (16). However, the underlying mechanisms have remained elusive for over 70 years. *E. coli*, the most widely used model for studying bacterial TLD, exhibits a brief resistance phase (approximately 1 h) in the absence of thymidine, during which CFU counts remain stable, followed by a rapid exponential death phase. This transient resistance is partly attributed to the use of intracellular dTDP-sugars as alternative thymidine sources, which temporarily support chromosomal DNA replication during thymidine starvation (44). In contrast, *Lm* exhibits a significantly prolonged resistance phase lasting up to 8 h (Fig. 2D). *E. coli* does not produce c-di-AMP, whereas *Lm* lacks the ability to utilize dTDP-sugars as precursors for DNA replication. Notably, reducing intracellular c-di-AMP levels in *Lm* Δ*thyA* abolishes the extended resistance phase, leading to a rapid loss of cell viability. These findings suggest that c-di-AMP may regulate an as-yet-unidentified pyrimidine salvage pathway in *Lm* that supplies thymidine during thymidine starvation, thereby sustaining the prolonged resistance phase. Although the mechanism by which c-di-AMP regulates TLD remains to be further studied, we have identified that the c-di-AMP-binding protein PstA, conserved in Firmicutes, serves as a negative regulator of this pathway. Moreover, PstA is found in several human pathogens, including *Lm*, *Staphylococcus aureus*, and *Enterococcus faecalis. S. aureus* thymidine auxotrophs, also known as thymidine-dependent small-colony variants, are frequently isolated from cystic fibrosis patients undergoing antifolate antibiotic treatment and are associated with more severe lung disease worldwide (45–47). Therefore, the role of PstA in regulating TLD may represent a clinically significant, conserved mechanism shared by pathogenic Firmicutes.

PstA belongs to the PII signaling protein family, which are multitasking regulators ubiquitously found across all domains of life. These proteins integrate signals of energy state and carbon/nitrogen balance by binding to various ligands, including nucleotides (ATP, ADP, and AMP), second messengers (cAMP and SAM-AMP), and metabolites (α-ketoglutarate and HCO_3_^−^). Different binding states, along with post-translational modifications, influence interactions with a wide range of effector proteins, such as transporters, metabolic enzymes, and transcription factors, thereby modulating various metabolic states of the cell (43). PII proteins contain two flexible loops: the T-loop and the B-loop. PstA is evolutionarily distinct from other PII family members. In PII proteins, the large T-loop mediates protein-protein interactions, whereas in PstA, this loop is shorter and contributes to c-di-AMP binding. Additionally, the B-loop in PstA is larger than in canonical PII proteins and lacks the highly conserved ATP-binding Walker A motif (40, 41). The binding of c-di-AMP induces significant changes in the position and orientation of the B-loop (40, 41), which may impact the interaction between PstA and its binding partners.

Although the downstream binding partners of PstA remain unknown, our findings, along with previous studies, suggest that PstA may act as a multitasking regulator of cell viability under various stress conditions, including antibiotic stress and nutrient starvation, and is detrimental to bacterial survival when c-di-AMP levels are low. *Lm* Δ*dacA* strain, in which c-di-AMP synthesis is abolished, exhibits significantly increased susceptibility to cefuroxime (33). Several studies have shown that deletion of *pstA* in Δ*dacA* restores cefuroxime resistance (33, 48). The bactericidal activity of β-lactam antibiotics under aerobic conditions is driven by the enhanced glycolytic flux they induce, which leads to an increased generation of ROS from the respiratory chain, ultimately resulting in cell death (49). Furthermore, the PstA-mediated increase in β-lactam susceptibility depends on the respiratory electron transport chain and is prominent only under aerobic conditions, with the effect largely diminished under hypoxia (48). These data suggest that the presence of PstA may lead to metabolic imbalance when c-di-AMP levels are low, which in turn impacts β-lactam susceptibility. Furthermore, Δ*dacA* exhibits increased cell lysis when grown in rich medium due to (p)ppGpp accumulation, which leads to metabolic imbalance and subsequent inactivation of the pleiotropic transcriptional regulator CodY (50), and depletion of *pstA* partially rescued this cell death (33, 41).

Our study demonstrated that Δ*thyA* exhibits significant intracellular growth defects, which lead to reduced bacterial burden in both murine intravenous and oral infection models. However, the growth defect was more pronounced following oral infection. In intravenous infections, *Lm* rapidly colonizes the liver, which serves as a major reservoir for dissemination (51). In contrast, during oral infection, *Lm* disseminates from the gastrointestinal tract to peripheral organs, including the gallbladder, which becomes one of the major primary bacterial reservoirs (39). Notably, our study found that Δ*thyA* exhibited a comparable ability to colonize the gastrointestinal tract as the WT strain. However, Δ*thyA* was unable to survive in the gallbladder. Similarly, defects in purine biosynthesis led to severely attenuated growth of *Lm* in *ex vivo* gallbladders and during intragastric infections (39, 52, 53). These findings suggest that bile may be limited in either pyrimidine or purine availability or that access to these nucleotides is restricted in this environment.

In summary, our study revealed a previously unrecognized role of c-di-AMP in modulating bacterial TLD. We anticipate that these findings will provide valuable insights into the physiological functions of the widespread bacterial second messenger c-di-AMP and broaden our understanding of its impact on antifolate antibiotic resistance and treatment outcomes in c-di-AMP-producing bacteria.

## MATERIALS AND METHODS

### Microbe strains and culturing conditions

*Escherichia coli* strains used for cloning were grown in LB at 37°C with shaking. *Lm* mutants were derived from the WT strain 10,403S (54). All *Lm* strains were cultured statically in BHI broth at 30°C unless otherwise stated. Antibiotics were used at the following concentrations: streptomycin, 200 µg/mL; chloramphenicol, 50 µg/mL (*E. coli*), 10 µg/mL (*Lm*); tetracycline, 2 µg/mL; ampicillin, 100 µg/mL; kanamycin, 50 µg/mL; nalidixic acid 25 μg/mL.

### Murine cell lines

The J2 virus-immortalized BMDM cell line was originally obtained from Dr. Dan Portnoy at the University of California, Berkeley. The iBMDMs were cultured at 37 °C in 5% CO2 in Dulbecco’s modified Eagle’s medium (DMEM) supplemented with 2 mM sodium glutamine, 1 mM sodium pyruvate, 10% heat-inactivated FBS, 0.1% 2-mercaptoethanol, and 10% L929 cell supernatants as a source of M-CSF.

### Plasmid and strain construction

The strains used in this study are listed in Table S2, and the primers are listed in Table S3. All *Lm* knockout mutants were generated by allelic exchange using the conjugation-proficient plasmid PliM, which expresses a mutated phenylalanyl-tRNA synthetase gene (pheS*). PheS* incorporates the toxic DL-4-chlorophenylalanine into cellular proteins, leading to bacterial cell death and enabling counterselection (55). Briefly, the PliM vector carrying 500 bp of upstream and downstream arms of the target gene was transferred to *E. coli* SM10 cells. The SM10 strain was mixed with the *Lm* strain and incubated for 6 h on BHI agar plates without antibiotics at 30°C for conjugation. Afterward, selection was performed on BHI plates containing both streptomycin and chloramphenicol to select against the *E. coli* and plasmid-free *Lm*. Single *Lm* colonies were passaged three times on BHI plates with streptomycin and chloramphenicol at 42°C to ensure plasmid integration. Single colonies were then passaged twice on BHI plates with 18 mM DL-4-chlorophenylalanine for allelic exchange. Overexpression strains were generated by integrating the pPL2 plasmid (56) into the *Lm* chromosome under the control of the p*Hyper* promoter (57) through conjugation, followed by selection on BHI plates supplemented with tetracycline. Complementation strains were generated by electroporating the pBAV1K-E plasmid (58) backbone carrying the genes under their native promoters, followed by selection on BHI plates supplemented with kanamycin. The resulting mutants were verified by Sanger sequencing and further confirmed by qRT-PCR.

### Antibiotic susceptibility assays

For the antibiotic susceptibility screenings, *Lm* culture in the mid-exponential phase was diluted with fresh BHI to an OD_600_ of 0.05. A 50 μL aliquot of the bacterial suspension was added to the BIOLOG microplates (PM11C and PM12B), sealed with an oxygen-permeable film, and incubated statically at 30°C for 6 h. The bacterial suspension was then serially diluted and plated on BHI plates for CFU enumeration.

For the disk diffusion assays, WT and Δ*dacA::disA* strains were grown statically in BHI with or without 0.5 mM IPTG supplementation at 30°C overnight. The bacterial cultures were adjusted to the same OD_600_, and 10 μL of the culture was added to 3 mL of melted top agar (0.8% agar in 0.8% NaCl solution with 0.5 mM IPTG, pre-cooled to 55°C), mixed by inversion, and then poured onto warm BHI agar plates. Once the plates had solidified, a 7 mm × 3 mm Whatman paper disk was placed on the top agar, and 5 μL of antibiotic solutions (100 mg/mL cephalothin; 60 mg/mL SXT; 100 mg/mL lincomycin; 50 mg/mL kanamycin) was applied to each disk. The plates were incubated at 30°C until the inhibition zone appeared.

### Bacterial growth and survival assays

The mid-exponential phase of *Lm* cultures was washed twice with PBS and pelleted by centrifugation. For the bacterial growth curve, the *Lm* pellet was resuspended in BHI to an OD₆₀₀ of 0.05 with various concentrations of thymidine. A 200 μL aliquot of the bacterial suspension was added to 96-well suspension plates sealed with oxygen-permeable film and cultured statically at 30°C. The OD₆₀₀ was measured every 30 min using a Microplate Reader (BioTek). For bacterial survival assays under thymidine starvation, the *Lm* pellet was resuspended in LB, which does not contain detectable levels of thymidine, to an OD₆₀₀ of 0.1 and cultured at 37°C with shaking. At various time points, the bacterial culture was serially diluted and plated on BHI plates supplemented with thymidine for CFU enumeration.

### Quantification of c-di-AMP

A 5 mL culture of *Lm* at the mid-exponential phase, grown in BHI under the indicated conditions, was collected by centrifugation (15,000 × *g*, 5 min), resuspended in 50 μL of Milli-Q water, and heat-killed at 95 °C for 30 min. The cells were then treated with lysozyme (0.2 mg/mL) and mutanolysin (50 U/mL) and incubated at 37°C for 30 min to lyse the cells. Subsequently, 500 μL of methanol was added to extract the nucleotides. The methanol extract was collected by centrifugation, and the bacterial pellet was washed with an additional 500 μL of methanol. Both methanol extracts were combined and dried at room temperature under the “V-AL” setting of a Vacufuge Concentrator (Eppendorf, USA) with caps open to allow complete evaporation of methanol. Each sample was then resuspended in water and used for c-di-AMP quantification by LC-MS/MS following a previously published protocol (19).

### Macrophage infections

For the study of intracellular growth of *Lm*, 0.5 × 10⁶ iBMDMs were plated in 24-well tissue culture plates and incubated overnight at 37°C with 5% CO₂. Mid-exponential phase *Lm* cultures grown in BHI containing 10 µg/mL thymidine were diluted 2,500- to 3,000-fold in BMM medium (DMEM GlutaMAX supplemented with 10% FBS and 10% L929 supernatants) to standardize bacterial concentrations. Macrophages were incubated with the *Lm* suspension at an MOI of 1. After a 0.5 h incubation, the macrophages were washed once with PBS and given fresh BMM medium containing 50 µg/mL gentamicin to eliminate extracellular bacteria. At various time points post-infection, macrophages were washed once with PBS and then lysed by adding H₂O, followed by incubation at 37°C for 10 min. Appropriate dilutions of the lysate were plated on BHI agar and incubated overnight at 37°C for *Lm* CFU enumeration. For the exogenous thymidine supplementation experiment, 100 µg/mL thymidine was added to the *Lm* suspension at the time of infection and maintained in the BMM medium throughout the experiment. For SXT susceptibility assays, WT and Δ*dacA::disA* strains grown in BHI with 0.5 mM IPTG were collected, washed twice with PBS, and used to infect iBMDMs at an MOI of 1. At 30 hpi, cells were washed and supplemented with fresh medium containing 50 µg/mL gentamicin and various concentrations of SXT. At 2, 4, and 6 hpi, cells were washed once and lysed for CFU enumeration. In the Δ*dacA::disA*+IPTG group, 0.5 mM IPTG was maintained in the BMM medium during infection to induce DisA expression. For the LDH assay, supernatants from macrophages treated with different concentrations of SXT or vehicle alone (DMSO) were collected to measure LDH release using the LDH-Glo Cytotoxicity Assay (Promega) according to the manufacturer’s instructions. The percentage of LDH release was normalized to macrophage lysis controls and the supernatants of untreated cells.

### Plaque assays

Murine L2 fibroblast cells were cultured in BMM medium (DMEM GlutaMAX supplemented with 10% fetal bovine serum). Plaque assays were performed on L2 fibroblast monolayers, as previously described (37), with the following modifications. A total of 1.2 × 10⁶ cells were seeded in 6-well tissue culture plates in 3 mL of BMM medium and grown overnight to form monolayers. The L2 monolayers were infected with *Lm* strains at an MOI of 0.2. After 1 h of incubation, extracellular *Lm* cells were washed off with PBS, and BMM medium containing 0.7% Superpure agarose (#G02PD-125), 10 µg/mL gentamicin, and varying concentrations of thymidine was preheated to 56°C and added to the cells. At 2 days post-infection, the staining solution (BMM medium containing 0.7% agarose and 0.25% neutral red) was added to the plates. After incubating for 18 h, the plaques were scanned using a gel imager and analyzed with ImageJ.

### Microscopic analysis

For the study of intracellular *Lm* morphology, 10^5^ iBMDMs were plated in 96-well poly-D-lysine-coated glass-bottom plates (MatTek Life Sciences, P96GC-1.5-5-F) and incubated overnight at 37°C with 5% CO₂. Macrophages were infected with *Lm* at an MOI of 10 or 20. After a 0.5-h incubation, the macrophages were washed once with PBS and given fresh BMM medium containing 50 µg/mL gentamicin to eliminate extracellular bacteria. At various time points post-infection, macrophages were washed twice with PBS and fixed in 4% paraformaldehyde in PBS for 15 min at room temperature. The cells were washed in Tris-buffered saline (TBS) with 0.1% Triton X-100 and blocked in TBS with 1% bovine serum albumin (BSA) at room temperature for 1 h. The *Lm* was stained with rabbit anti-*Lm* antibody (Abnova, PAB29797) at a 1:200 dilution in TBS with 1% BSA overnight at 4°C. The macrophages were washed five times with 200 µL TBST buffer and incubated with Alexa Fluor 488-conjugated goat anti-rabbit IgG secondary antibody (Invitrogen, A11008) at a 1:200 dilution, Alexa Fluor 568 phalloidin (Invitrogen, A12380) at a 1:1,000 dilution, and DAPI at a 1:1,000 dilution in TBS with 1% BSA for 2 h at 4°C. The cells were then washed five times with 200 µL TBST buffer, maintained in TBS, and visualized under oil immersion at 100× using a Nikon Eclipse fluorescence microscope.

For the propidium iodide staining of bacteria, mid-exponential phase *Lm* cultures were washed twice with sterile PBS and diluted with fresh LB to an OD_600_ of 0.1, supplemented with 0.1 mg/mL propidium iodide. A 5 μL aliquot of the bacterial suspension was placed onto an agarose gel pad (1% agarose in LB) and air-dried. The agar pad was then covered with a coverslip and sealed with glue. The bacteria were visualized under oil immersion at 100× magnification using a Nikon Eclipse microscope.

### Mouse infection

All the mice used in this study were of the C57BL/6J background and purchased from Jackson Laboratories. The mice were maintained under SPF conditions, as ensured by the rodent health monitoring program overseen by the Animal Care Facility of the Shimadzu Institute for Research Technologies at the University of Texas at Arlington. All experiments were carried out using mice aged 7–10 weeks, matched by gender, age, and body weight.

Prior to animal experiments, *Lm* strains were cultured overnight statically at 30°C in BHI, then back-diluted 1:5 in fresh BHI, and grown for 1 h at 37°C with shaking. The bacterial suspension was diluted with cold PBS. For systematic infection, a 200 μL aliquot of the *Lm* suspension was injected R.O. into the mice with an inoculum of 10^5^ CFU/mouse. For the oral infection, mice will be given 5 mg/mL streptomycin in water with regular food for 48 h, followed by a 16-h fasting. The mice were orally instilled with 20 μL of *Lm* suspension in PBS via pipetting, with an inoculum of 10^8^ CFU/mouse. After infection, the infected mice will be provided with regular food and water. At 72 hpi, the mice from both infection models were euthanized by CO_2_, and the liver, spleen, gallbladder, cecum, small intestine, colon, and feces were collected. The liver, spleen, cecum, small intestine, and colon were homogenized and lysed in 10 mL of lysis buffer (0.1% IPEGAL in water) using a Tissue Tearor Homogenizer. The gallbladder was opened using a sterile wooden stick in 500 μL of lysis buffer, and fecal samples were mixed in 1 mL of lysis buffer by vortexing. Bacterial burdens were enumerated by plating serial dilutions on BHI plates containing streptomycin and 10 μg/mL thymidine to support the growth of Δ*thyA* mutants. For intestinal and fecal samples, an additional 50 μg/mL nalidixic acid was added to prevent contamination from the microbiota.

### Quantification and statistical analysis

All numerical data were statistically analyzed and plotted using GraphPad Prism software. For bacterial growth curves, survival assays, tissue culture experiments, and c-di-AMP quantification plots, mean values of biological replicates are plotted, with error bars indicating ±SD. Data shown in animal infection plots are represented as the median of biological replicates. The exact numbers of replicates are stated in the figure legends. Statistical tests used and the corresponding statistical parameters for each experiment are detailed in their respective figure legends. All experiments were repeated at least twice. No methods were employed to determine whether the data met the assumptions of the statistical approach.

## AFTER EPUB

*[This article was published on 11 December 2025 with the incorrect Video S2. The supplemental material was corrected in the current version, posted on 17 December 2025.]*

## SUPPLEMENTAL MATERIAL

Supplemental Figures mbio.03351-25-s0001.pdf Figures S1-S4.

Supplemental Tables mbio.03351-25-s0002.pdf Tables S1-S3.

Video S1 mbio.03351-25-s0003.mp4 Δ*thyA* strain stained with propidium iodide.

Video S2 mbio.03351-25-s0004.mp4 Δ*thyA::pdeA* strain stained with propidium iodide.

ASM does not own the copyrights to Supplemental Material that may be linked to, or accessed through, an article. The authors have granted ASM a non-exclusive, world-wide license to publish the Supplemental Material files. Please contact the corresponding author directly for reuse.
