# Supplementary material for: Cyclic di-AMP inhibits Listeria monocytogenes thymineless death during infection
Source: mBio. 2025 Dec 11;17(1):e03351-25. doi: 10.1128/mbio.03351-25 (PMC12802241; doi:10.1128/mbio.03351-25)
Supplement: Supplemental Figures — Figures S1-S4. [file mbio.03351-25-s0001.pdf]

Supplemental Materials

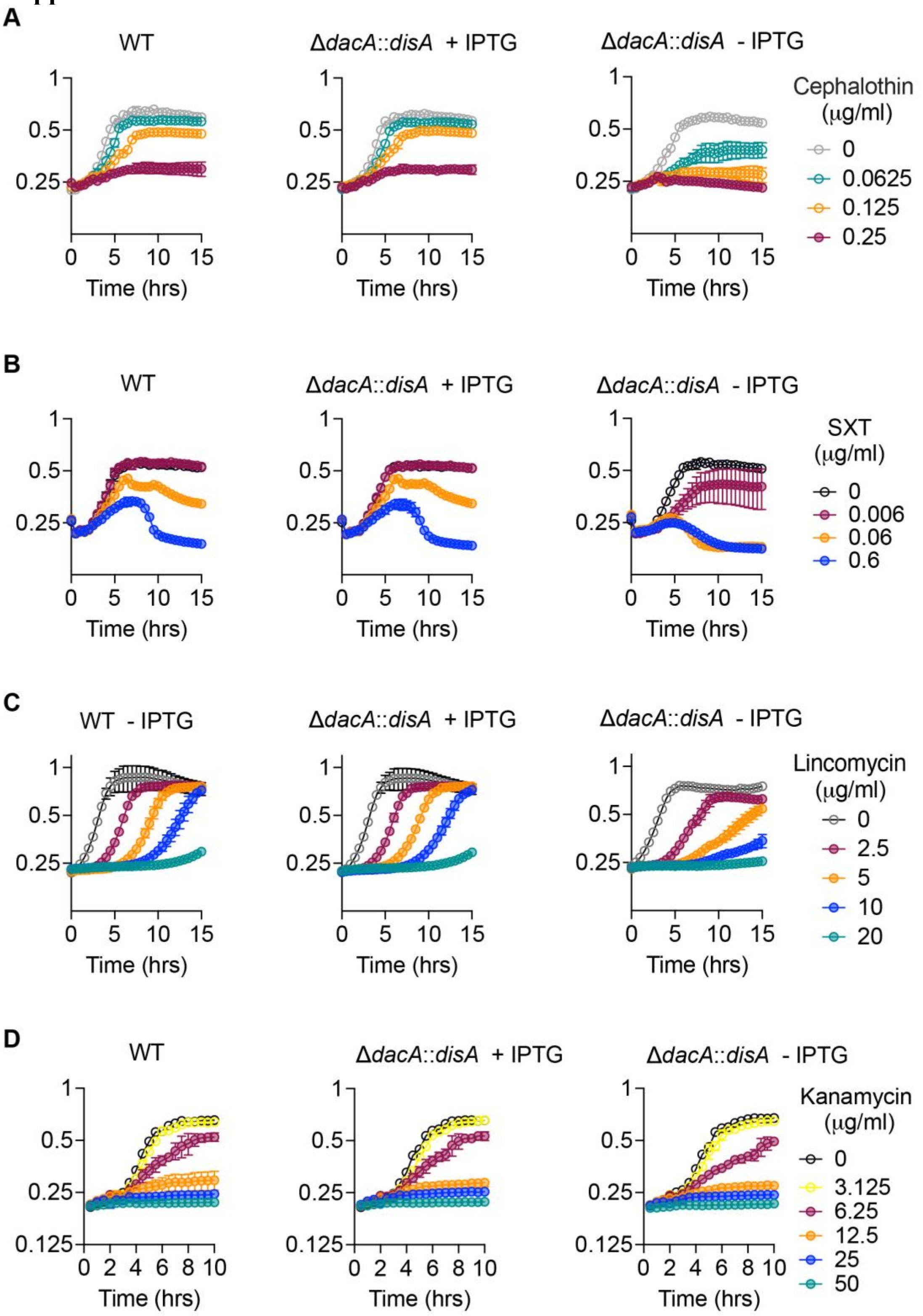

Supplemental Figure 1. Bacterial growth in BHI broth supplemented with different concentrations of antibiotics. The OD at 600 nm was measured using a microplate reader, and IPTG (0.5 mM) was supplemented as indicated.

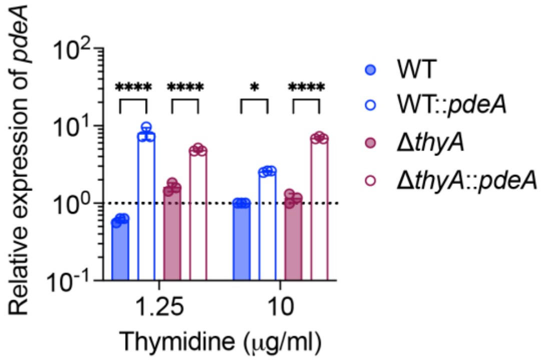

Supplemental Figure 2. The expression of *pdeA* as measured by qRT-PCR. The *pdeA* mRNA level in each strain was normalized to that of the WT strain grown in BHI with 10 μg/mL thymidine, using 16S rRNA as the internal control. Mean values of biological triplicates are plotted, and error bars indicate ±SD. *P* values were calculated using two-way ANOVA analysis. Asterisks indicate that differences are statistically significant (*, $P < 0.05$; ****, $P < 0.0001$), and "ns" indicates no significant difference.

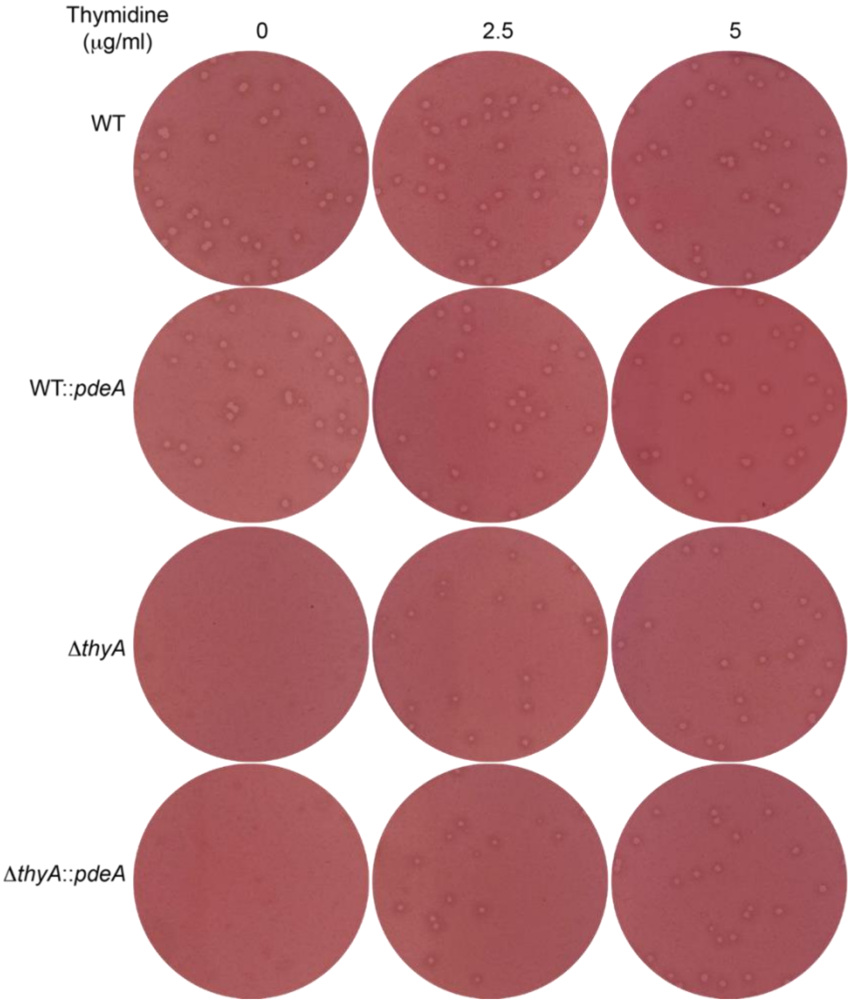

Supplemental Figure 3. **Plaques in L2 fibroblasts infected with WT, WT::*pdeA*, Δ*thyA*, and Δ*thyA*::*pdeA* strain strains**. The L2 monolayers were infected with *Lm* strains at an MOI of 0.2. After 1 hour of incubation, extracellular *Lm* cells were washed off with PBS, and BMM medium containing 0.7% Superpure agarose (#G02PD-125), 10 μg/mL gentamicin, and varying concentrations of thymidine was preheated to 56°C and added to the cells. At 2 days post-infection, the staining solution (BMM medium containing 0.7% agarose and 0.25% neutral red) was added and incubated for 18 hours before the plates were scanned.

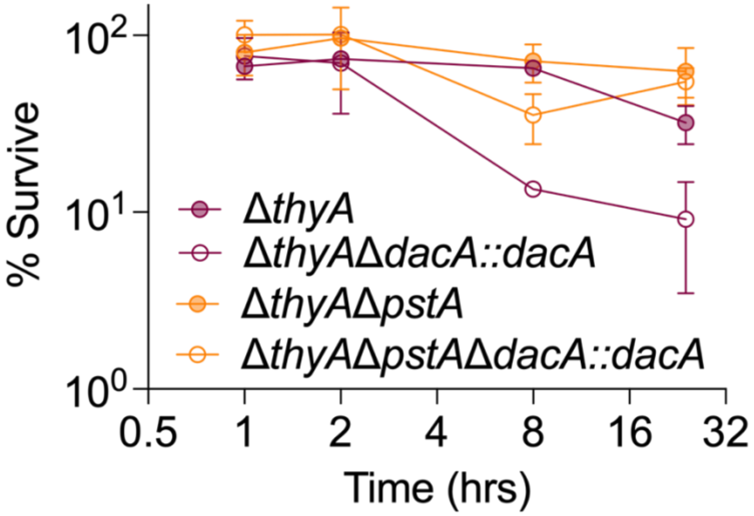

Supplemental Figure 4. **Bacterial survival in the absence of thymidine**. The strains were incubated in LB broth, and CFUs were enumerated by plating the bacteria on BHI agar plates with thymidine at each time point. The percentage of survival at each time point was calculated by normalizing the CFU count to the value at time 0.
