# Supplementary material for: Cyclic di-AMP inhibits Listeria monocytogenes thymineless death during infection
Source: mBio. 2025 Dec 11;17(1):e03351-25. doi: 10.1128/mbio.03351-25 (PMC12802241; doi:10.1128/mbio.03351-25)
Supplement: Supplemental Tables — Tables S1-S3. [file mbio.03351-25-s0002.pdf]

Supplemental Table 1. Relative antibiotic susceptibility of Δ*dacA*::*disA* compared to WT

Well	Antibiotics	WT (CFU/mL)		Δ*dacA*::*disA* (CFU/mL)		Inhibition fold*		Relative inhibition fold	
		Untreated	Antibiotic _Treated	Untreated	Antibiotic _Treated	WT	Δ*dacA*::*disA*	Δ*dacA*::*disA*/WT	log2 (Relative Inhibition fold)
A1	Penicillin G	1.67E+09	1.80E+09	2.00E+08	7.50E+07	108.0	37.5	0.3	-0.7
A2	Penicillin G	1.67E+09	4.30E+07	2.00E+08	2.40E+06	2.6	1.2	0.5	-0.9
A3	Penicillin G	1.67E+09	4.10E+07	2.00E+08	2.70E+06	2.5	1.4	0.5	-1.2
A4	Penicillin G	1.67E+09	4.00E+07	2.00E+08	2.20E+06	2.4	1.1	0.5	-0.9
A5	Tetracycline	1.67E+09	3.50E+09	2.00E+08	2.40E+08	210.0	120.0	0.6	-1.2
A6	Tetracycline	1.67E+09	2.60E+09	2.00E+08	1.70E+08	156.0	85.0	0.5	-1.1
A7	Tetracycline	1.67E+09	1.20E+09	2.00E+08	1.40E+08	72.0	70.0	1.0	-24.6
A8	Tetracycline	1.67E+09	5.60E+08	2.00E+08	2.20E+07	33.6	11.0	0.3	-0.6
A9	Carbenicillin	1.67E+09	3.90E+09	2.00E+08	1.50E+08	234.0	75.0	0.3	-0.6
A10	Carbenicillin	1.67E+09	1.50E+09	2.00E+08	1.60E+08	90.0	80.0	0.9	-5.9
A11	Carbenicillin	1.67E+09	5.20E+08	2.00E+08	2.90E+06	31.2	1.5	0.0	-0.2
A12	Carbenicillin	1.67E+09	6.00E+07	2.00E+08	3.60E+06	3.6	1.8	0.5	-1.0
B1	Oxacillin	1.67E+09	5.10E+07	2.00E+08	2.40E+06	3.1	1.2	0.4	-0.7
B2	Oxacillin	1.67E+09	2.90E+07	2.00E+08	2.50E+06	1.7	1.3	0.7	-2.1
B3	Oxacillin	1.67E+09	4.70E+07	2.00E+08	2.50E+06	2.8	1.3	0.4	-0.9
B4	Oxacillin	1.67E+09	5.30E+07	2.00E+08	1.30E+06	3.2	0.7	0.2	-0.4
B5	Penimepicycline	1.67E+09	2.50E+09	2.00E+08	1.20E+08	150.0	60.0	0.4	-0.8
B6	Penimepicycline	1.67E+09	2.30E+09	2.00E+08	7.00E+07	138.0	35.0	0.3	-0.5
B7	Penimepicycline	1.67E+09	1.30E+08	2.00E+08	1.60E+07	7.8	8.0	1.0	27.4
B8	Penimepicycline	1.67E+09	3.00E+07	2.00E+08	1.60E+07	1.8	8.0	4.4	0.5
B9	Polymyxin B	1.67E+09	1.40E+09	2.00E+08	2.50E+08	84.0	125.0	1.5	1.7
B10	Polymyxin B	1.67E+09	2.10E+09	2.00E+08	2.10E+08	126.0	105.0	0.8	-3.8
B11	Polymyxin B	1.67E+09	1.80E+09	2.00E+08	2.60E+08	108.0	130.0	1.2	3.7
B12	Polymyxin B	1.67E+09	1.60E+09	2.00E+08	2.70E+08	96.0	135.0	1.4	2.0
C1	Paromomycin	1.67E+09	2.80E+09	2.00E+08	1.47E+08	168.0	73.5	0.4	-0.8

C2	Paromomycin	1.67E+09	2.40E+09	2.00E+08	1.35E+08	144.0	67.5	0.5	-0.9
C3	Paromomycin	1.67E+09	2.30E+09	2.00E+08	1.14E+08	138.0	57.0	0.4	-0.8
C4	Paromomycin	1.67E+09	2.20E+09	2.00E+08	8.30E+07	132.0	41.5	0.3	-0.6
C5	Vancomycin	1.67E+09	1.30E+07	2.00E+08	6.80E+06	0.8	3.4	4.4	0.5
C6	Vancomycin	1.67E+09	1.60E+07	2.00E+08	5.40E+06	1.0	2.7	2.8	0.7
C7	Vancomycin	1.67E+09	3.40E+07	2.00E+08	6.70E+06	2.0	3.4	1.6	1.4
C8	Vancomycin	1.67E+09	3.50E+07	2.00E+08	7.20E+06	2.1	3.6	1.7	1.3
C9	D,L-Serine hydroxamate	1.67E+09	3.10E+09	2.00E+08	1.40E+08	186.0	70.0	0.4	-0.7
C10	D,L-Serine hydroxamate	1.67E+09	6.20E+06	2.00E+08	8.00E+04	0.4	0.0	0.1	-0.3
C11	D,L-Serine hydroxamate	1.67E+09	0.00E+00	2.00E+08	0.00E+00	0.0	0.0	ND	ND
C12	D,L-Serine hydroxamate	1.67E+09	0.00E+00	2.00E+08	0.00E+00	0.0	0.0	ND	ND
D1	Sisomicin	1.67E+09	2.60E+09	2.00E+08	1.11E+08	156.0	55.5	0.4	-0.7
D2	Sisomicin	1.67E+09	2.20E+09	2.00E+08	1.19E+08	132.0	59.5	0.5	-0.9
D3	Sisomicin	1.67E+09	3.00E+09	2.00E+08	1.27E+08	180.0	63.5	0.4	-0.7
D4	Sisomicin	1.67E+09	2.90E+09	2.00E+08	9.20E+07	174.0	46.0	0.3	-0.5
D5	Sulfamethazine	1.67E+09	2.70E+09	2.00E+08	1.30E+08	162.0	65.0	0.4	-0.8
D6	Sulfamethazine	1.67E+09	4.40E+09	2.00E+08	1.60E+08	264.0	80.0	0.3	-0.6
D7	Sulfamethazine	1.67E+09	6.10E+09	2.00E+08	2.40E+08	366.0	120.0	0.3	-0.6
D8	Sulfamethazine	1.67E+09	3.60E+09	2.00E+08	1.48E+08	216.0	74.0	0.3	-0.6
D9	Novobiocin	1.67E+09	2.20E+07	2.00E+08	1.40E+07	1.3	7.0	5.3	0.4
D10	Novobiocin	1.67E+09	3.10E+07	2.00E+08	2.20E+07	1.9	11.0	5.9	0.4
D11	Novobiocin	1.67E+09	1.20E+07	2.00E+08	2.70E+07	0.7	13.5	18.8	0.2
D12	Novobiocin	1.67E+09	2.20E+07	2.00E+08	1.60E+07	1.3	8.0	6.1	0.4
E1	2,4-Diamino-6,7-diisopropylpteridine	1.67E+09	2.90E+05	2.00E+08	2.20E+06	0.0	1.1	63.2	0.2
E2	2,4-Diamino-6,7-diisopropylpteridine	1.67E+09	4.00E+04	2.00E+08	2.10E+06	0.0	1.1	437.5	0.1
E3	2,4-Diamino-6,7-diisopropylpteridine	1.67E+09	0.00E+00	2.00E+08	4.00E+05	0.0	0.2	ND	ND

E4	2,4-Diamino-6,7-diisopropylpteridine	1.67E+09	0.00E+00	2.00E+08	0.00E+00	0.0	0.0	ND	ND
E5	Sulfadiazine	1.67E+09	3.60E+09	2.00E+08	1.90E+08	216.0	95.0	0.4	-0.8
E6	Sulfadiazine	1.67E+09	4.40E+09	2.00E+08	2.10E+08	264.0	105.0	0.4	-0.8
E7	Sulfadiazine	1.67E+09	3.10E+09	2.00E+08	1.50E+08	186.0	75.0	0.4	-0.8
E8	Sulfadiazine	1.67E+09	4.00E+09	2.00E+08	1.30E+08	240.0	65.0	0.3	-0.5
E9	Benzethonium chloride	1.67E+09	1.30E+05	2.00E+08	8.10E+05	0.0	0.4	51.9	0.2
E10	Benzethonium chloride	1.67E+09	0.00E+00	2.00E+08	0.00E+00	0.0	0.0	ND	ND
E11	Benzethonium chloride	1.67E+09	0.00E+00	2.00E+08	0.00E+00	0.0	0.0	ND	ND
E12	Benzethonium chloride	1.67E+09	0.00E+00	2.00E+08	0.00E+00	0.0	0.0	ND	ND
F1	Tobramycin	1.67E+09	2.70E+09	2.00E+08	1.33E+08	162.0	66.5	0.4	-0.8
F2	Tobramycin	1.67E+09	4.30E+09	2.00E+08	1.13E+08	258.0	56.5	0.2	-0.5
F3	Tobramycin	1.67E+09	2.80E+09	2.00E+08	1.10E+08	168.0	55.0	0.3	-0.6
F4	Tobramycin	1.67E+09	4.80E+09	2.00E+08	6.60E+07	288.0	33.0	0.1	-0.3
F5	Sulfathiazole	1.67E+09	2.50E+09	2.00E+08	1.90E+08	150.0	95.0	0.6	-1.5
F6	Sulfathiazole	1.67E+09	4.50E+09	2.00E+08	2.40E+08	270.0	120.0	0.4	-0.9
F7	Sulfathiazole	1.67E+09	2.40E+09	2.00E+08	1.01E+08	144.0	50.5	0.4	-0.7
F8	Sulfathiazole	1.67E+09	2.60E+09	2.00E+08	9.30E+07	156.0	46.5	0.3	-0.6
F9	5-Fluoroorotic acid	1.67E+09	2.10E+09	2.00E+08	1.70E+08	126.0	85.0	0.7	-1.8
F10	5-Fluoroorotic acid	1.67E+09	3.10E+09	2.00E+08	2.50E+08	186.0	125.0	0.7	-1.7
F11	5-Fluoroorotic acid	1.67E+09	4.40E+09	2.00E+08	1.90E+08	264.0	95.0	0.4	-0.7
F12	5-Fluoroorotic acid	1.67E+09	3.70E+09	2.00E+08	1.80E+08	222.0	90.0	0.4	-0.8
G1	Spectinomycin	1.67E+09	3.20E+09	2.00E+08	9.60E+07	192.0	48.0	0.3	-0.5
G2	Spectinomycin	1.67E+09	3.00E+09	2.00E+08	1.30E+08	180.0	65.0	0.4	-0.7
G3	Spectinomycin	1.67E+09	4.30E+09	2.00E+08	1.20E+08	258.0	60.0	0.2	-0.5
G4	Spectinomycin	1.67E+09	3.60E+09	2.00E+08	8.70E+07	216.0	43.5	0.2	-0.4
G5	Sulfamethoxazole	1.67E+09	3.20E+09	2.00E+08	1.30E+08	192.0	65.0	0.3	-0.6
G6	Sulfamethoxazole	1.67E+09	4.10E+09	2.00E+08	1.15E+08	246.0	57.5	0.2	-0.5

G7	Sulfamethoxazole	1.67E+09	2.80E+09	2.00E+08	1.05E+08	168.0	52.5	0.3	-0.6
G8	Sulfamethoxazole	1.67E+09	3.50E+09	2.00E+08	1.00E+08	210.0	50.0	0.2	-0.5
G9	L-Aspartic-β-hydroxamate	1.67E+09	4.20E+09	2.00E+08	1.30E+08	252.0	65.0	0.3	-0.5
G10	L-Aspartic-β-hydroxamate	1.67E+09	3.30E+09	2.00E+08	1.04E+08	198.0	52.0	0.3	-0.5
G11	L-Aspartic-β-hydroxamate	1.67E+09	2.10E+09	2.00E+08	9.70E+07	126.0	48.5	0.4	-0.7
G12	L-Aspartic-β-hydroxamate	1.67E+09	3.40E+09	2.00E+08	7.70E+07	204.0	38.5	0.2	-0.4
H1	Spiramycin	1.67E+09	2.70E+09	2.00E+08	8.60E+07	162.0	43.0	0.3	-0.5
H2	Spiramycin	1.67E+09	3.50E+07	2.00E+08	2.10E+06	2.1	1.1	0.5	-1.0
H3	Spiramycin	1.67E+09	3.90E+07	2.00E+08	2.50E+06	2.3	1.3	0.5	-1.1
H4	Spiramycin	1.67E+09	2.60E+07	2.00E+08	1.90E+06	1.6	1.0	0.6	-1.4
H5	Rifampicin	1.67E+09	2.70E+09	2.00E+08	4.00E+06	162.0	2.0	0.0	-0.2
H6	Rifampicin	1.67E+09	2.90E+07	2.00E+08	3.40E+06	1.7	1.7	1.0	-29.8
H7	Rifampicin	1.67E+09	2.60E+07	2.00E+08	2.50E+06	1.6	1.3	0.8	-3.1
H8	Rifampicin	1.67E+09	4.70E+07	2.00E+08	3.10E+06	2.8	1.6	0.5	-1.2
H9	Dodecyltrimethyl ammonium	1.67E+09	1.50E+08	2.00E+08	1.11E+07	9.0	5.6	0.6	-1.4
H10	Dodecyltrimethyl ammonium	1.67E+09	5.30E+06	2.00E+08	1.14E+06	0.3	0.6	1.8	1.2
H11	Dodecyltrimethyl ammonium	1.67E+09	0.00E+00	2.00E+08	0.00E+00	0.0	0.0	ND	ND
H12	Dodecyltrimethyl ammonium	1.67E+09	0.00E+00	2.00E+08	0.00E+00	0.0	0.0	ND	ND
A1	Amikacin	1.93E+09	1.50E+09	6.77E+07	5.80E+07	77.6	85.7	1.1	7.0
A2	Amikacin	1.93E+09	1.31E+09	6.77E+07	5.70E+07	67.8	84.2	1.2	3.2
A3	Amikacin	1.93E+09	1.68E+09	6.77E+07	5.50E+07	86.9	81.3	0.9	-10.4
A4	Amikacin	1.93E+09	1.30E+09	6.77E+07	5.10E+07	67.2	75.4	1.1	6.1
A5	Chlortetracycline	1.93E+09	2.90E+07	6.77E+07	4.80E+06	1.5	7.1	4.7	0.4
A6	Chlortetracycline	1.93E+09	2.30E+07	6.77E+07	5.20E+06	1.2	7.7	6.5	0.4
A7	Chlortetracycline	1.93E+09	7.50E+06	6.77E+07	5.80E+06	0.4	8.6	22.1	0.2

A8	Chlortetracycline	1.93E+09	1.40E+06	6.77E+07	3.20E+06	0.1	4.7	65.3	0.2
A9	Lincomycin	1.93E+09	1.70E+07	6.77E+07	3.40E+06	0.9	5.0	5.7	0.4
A10	Lincomycin	1.93E+09	2.10E+07	6.77E+07	3.20E+06	1.1	4.7	4.4	0.5
A11	Lincomycin	1.93E+09	1.60E+07	6.77E+07	4.10E+05	0.8	0.6	0.7	-2.2
A12	Lincomycin	1.93E+09	1.00E+06	6.77E+07	1.00E+02	0.1	0.0	0.0	-0.1
B1	Amoxicillin	1.93E+09	2.80E+07	6.77E+07	2.10E+06	1.4	3.1	2.1	0.9
B2	Amoxicillin	1.93E+09	1.60E+07	6.77E+07	1.24E+06	0.8	1.8	2.2	0.9
B3	Amoxicillin	1.93E+09	1.50E+07	6.77E+07	1.24E+06	0.8	1.8	2.4	0.8
B4	Amoxicillin	1.93E+09	1.80E+07	6.77E+07	1.44E+06	0.9	2.1	2.3	0.8
B5	Cloxacillin	1.93E+09	2.00E+07	6.77E+07	3.30E+06	1.0	4.9	4.7	0.4
B6	Cloxacillin	1.93E+09	1.60E+07	6.77E+07	1.20E+06	0.8	1.8	2.1	0.9
B7	Cloxacillin	1.93E+09	2.10E+07	6.77E+07	6.60E+05	1.1	1.0	0.9	-6.4
B8	Cloxacillin	1.93E+09	3.20E+07	6.77E+07	1.40E+05	1.7	0.2	0.1	-0.3
B9	Lomefloxacin	1.93E+09	1.20E+09	6.77E+07	5.70E+07	62.1	84.2	1.4	2.3
B10	Lomefloxacin	1.93E+09	1.60E+09	6.77E+07	5.10E+07	82.8	75.4	0.9	-7.4
B11	Lomefloxacin	1.93E+09	1.30E+09	6.77E+07	5.90E+07	67.2	87.2	1.3	2.7
B12	Lomefloxacin	1.93E+09	2.10E+09	6.77E+07	6.80E+07	108.6	100.5	0.9	-8.9
C1	Bleomycin	1.93E+09	2.20E+09	6.77E+07	6.80E+07	113.8	100.5	0.9	-5.6
C2	Bleomycin	1.93E+09	1.60E+09	6.77E+07	7.70E+07	82.8	113.8	1.4	2.2
C3	Bleomycin	1.93E+09	1.59E+09	6.77E+07	9.00E+07	82.2	133.0	1.6	1.4
C4	Bleomycin	1.93E+09	2.10E+09	6.77E+07	7.60E+07	108.6	112.3	1.0	20.7
C5	Colistin	1.93E+09	1.60E+09	6.77E+07	7.00E+07	82.8	103.4	1.2	3.1
C6	Colistin	1.93E+09	1.60E+09	6.77E+07	6.20E+07	82.8	91.6	1.1	6.8
C7	Colistin	1.93E+09	1.90E+09	6.77E+07	6.90E+07	98.3	102.0	1.0	18.8
C8	Colistin	1.93E+09	2.50E+09	6.77E+07	7.00E+07	129.3	103.4	0.8	-3.1
C9	Minocycline	1.93E+09	9.10E+07	6.77E+07	5.10E+06	4.7	7.5	1.6	1.5
C10	Minocycline	1.93E+09	1.70E+07	6.77E+07	3.00E+06	0.9	4.4	5.0	0.4
C11	Minocycline	1.93E+09	1.10E+07	6.77E+07	1.60E+06	0.6	2.4	4.2	0.5
C12	Minocycline	1.93E+09	2.00E+07	6.77E+07	4.00E+06	1.0	5.9	5.7	0.4

D1	Capreomycin	1.93E+09	1.12E+09	6.77E+07	6.60E+07	57.9	97.5	1.7	1.3
D2	Capreomycin	1.93E+09	1.20E+09	6.77E+07	4.60E+07	62.1	68.0	1.1	7.6
D3	Capreomycin	1.93E+09	1.31E+09	6.77E+07	4.80E+07	67.8	70.9	1.0	15.1
D4	Capreomycin	1.93E+09	1.20E+09	6.77E+07	3.60E+07	62.1	53.2	0.9	-4.5
D5	Demeclocycline	1.93E+09	2.70E+08	6.77E+07	5.80E+06	14.0	8.6	0.6	-1.4
D6	Demeclocycline	1.93E+09	1.30E+07	6.77E+07	4.00E+06	0.7	5.9	8.8	0.3
D7	Demeclocycline	1.93E+09	1.50E+07	6.77E+07	6.20E+06	0.8	9.2	11.8	0.3
D8	Demeclocycline	1.93E+09	1.40E+07	6.77E+07	7.30E+06	0.7	10.8	14.9	0.3
D9	Nafcillin	1.93E+09	2.70E+07	6.77E+07	2.00E+06	1.4	3.0	2.1	0.9
D10	Nafcillin	1.93E+09	2.00E+07	6.77E+07	9.00E+05	1.0	1.3	1.3	2.8
D11	Nafcillin	1.93E+09	2.60E+07	6.77E+07	1.50E+05	1.3	0.2	0.2	-0.4
D12	Nafcillin	1.93E+09	4.00E+07	6.77E+07	1.00E+02	2.1	0.0	0.0	-0.1
E1	Cefazolin	1.93E+09	2.20E+09	6.77E+07	9.20E+07	113.8	136.0	1.2	3.9
E2	Cefazolin	1.93E+09	2.20E+09	6.77E+07	6.00E+07	113.8	88.7	0.8	-2.8
E3	Cefazolin	1.93E+09	8.00E+08	6.77E+07	2.50E+06	41.4	3.7	0.1	-0.3
E4	Cefazolin	1.93E+09	2.50E+07	6.77E+07	2.90E+06	1.3	4.3	3.3	0.6
E5	Enoxacin	1.93E+09	2.70E+09	6.77E+07	8.70E+07	139.7	128.6	0.9	-8.4
E6	Enoxacin	1.93E+09	2.20E+09	6.77E+07	8.30E+07	113.8	122.7	1.1	9.2
E7	Enoxacin	1.93E+09	2.50E+09	6.77E+07	1.15E+08	129.3	170.0	1.3	2.5
E8	Enoxacin	1.93E+09	1.40E+09	6.77E+07	7.10E+07	72.4	104.9	1.4	1.9
E9	Nalidixic acid	1.93E+09	1.80E+09	6.77E+07	8.40E+07	93.1	124.1	1.3	2.4
E10	Nalidixic acid	1.93E+09	2.20E+09	6.77E+07	9.10E+07	113.8	134.5	1.2	4.1
E11	Nalidixic acid	1.93E+09	2.10E+09	6.77E+07	8.20E+07	108.6	121.2	1.1	6.3
E12	Nalidixic acid	1.93E+09	2.50E+09	6.77E+07	6.50E+07	129.3	96.1	0.7	-2.3
F1	Chloramphenicol	1.93E+09	5.80E+08	6.77E+07	8.00E+07	30.0	118.2	3.9	0.5
F2	Chloramphenicol	1.93E+09	5.20E+08	6.77E+07	4.00E+07	26.9	59.1	2.2	0.9
F3	Chloramphenicol	1.93E+09	1.40E+07	6.77E+07	4.10E+06	0.7	6.1	8.4	0.3
F4	Chloramphenicol	1.93E+09	1.21E+07	6.77E+07	2.30E+06	0.6	3.4	5.4	0.4
F5	Erythromycin	1.93E+09	2.20E+07	6.77E+07	3.40E+06	1.1	5.0	4.4	0.5

F6	Erythromycin	1.93E+09	1.50E+07	6.77E+07	3.20E+06	0.8	4.7	6.1	0.4
F7	Erythromycin	1.93E+09	1.03E+07	6.77E+07	2.20E+06	0.5	3.3	6.1	0.4
F8	Erythromycin	1.93E+09	1.06E+07	6.77E+07	2.20E+06	0.5	3.3	5.9	0.4
F9	Neomycin	1.93E+09	2.50E+09	6.77E+07	9.50E+07	129.3	140.4	1.1	8.4
F10	Neomycin	1.93E+09	1.80E+09	6.77E+07	1.02E+08	93.1	150.7	1.6	1.4
F11	Neomycin	1.93E+09	2.80E+09	6.77E+07	8.00E+07	144.8	118.2	0.8	-3.4
F12	Neomycin	1.93E+09	1.40E+09	6.77E+07	5.70E+07	72.4	84.2	1.2	4.6
G1	Ceftriaxone	1.93E+09	2.40E+09	6.77E+07	2.10E+08	124.1	310.3	2.5	0.8
G2	Ceftriaxone	1.93E+09	2.20E+09	6.77E+07	1.33E+08	113.8	196.6	1.7	1.3
G3	Ceftriaxone	1.93E+09	2.60E+09	6.77E+07	1.07E+08	134.5	158.1	1.2	4.3
G4	Ceftriaxone	1.93E+09	1.60E+09	6.77E+07	9.60E+07	82.8	141.9	1.7	1.3
G5	Gentamicin	1.93E+09	2.80E+09	6.77E+07	1.34E+08	144.8	198.0	1.4	2.2
G6	Gentamicin	1.93E+09	2.30E+09	6.77E+07	1.13E+08	119.0	167.0	1.4	2.0
G7	Gentamicin	1.93E+09	2.00E+09	6.77E+07	1.36E+08	103.4	201.0	1.9	1.0
G8	Gentamicin	1.93E+09	2.10E+09	6.77E+07	7.70E+07	108.6	113.8	1.0	14.9
G9	Potassium tellurite	1.93E+09	2.50E+09	6.77E+07	9.90E+07	129.3	146.3	1.1	5.6
G10	Potassium tellurite	1.93E+09	2.40E+09	6.77E+07	2.00E+08	124.1	295.6	2.4	0.8
G11	Potassium tellurite	1.93E+09	2.00E+09	6.77E+07	1.80E+08	103.4	266.0	2.6	0.7
G12	Potassium tellurite	1.93E+09	3.30E+09	6.77E+07	2.10E+08	170.7	310.3	1.8	1.2
H1	Cephalothin	1.93E+09	1.90E+09	6.77E+07	1.26E+07	98.3	18.6	0.2	-0.4
H2	Cephalothin	1.93E+09	1.50E+09	6.77E+07	3.00E+06	77.6	4.4	0.1	-0.2
H3	Cephalothin	1.93E+09	2.80E+07	6.77E+07	2.80E+06	1.4	4.1	2.9	0.7
H4	Cephalothin	1.93E+09	1.90E+07	6.77E+07	1.90E+06	1.0	2.8	2.9	0.7
H5	Kanamycin	1.93E+09	2.10E+09	6.77E+07	1.12E+08	108.6	165.5	1.5	1.6
H6	Kanamycin	1.93E+09	1.50E+09	6.77E+07	1.23E+08	77.6	181.8	2.3	0.8
H7	Kanamycin	1.93E+09	1.70E+09	6.77E+07	9.20E+07	87.9	136.0	1.5	1.6
H8	Kanamycin	1.93E+09	2.60E+08	6.77E+07	5.50E+07	13.4	81.3	6.0	0.4
H9	Ofloxacin	1.93E+09	2.60E+09	6.77E+07	1.30E+08	134.5	192.1	1.4	1.9
H10	Ofloxacin	1.93E+09	2.70E+09	6.77E+07	1.80E+08	139.7	266.0	1.9	1.1

H11	Ofloxacin	1.93E+09	1.50E+09	6.77E+07	1.80E+08	77.6	266.0	3.4	0.6
H12	Ofloxacin	1.93E+09	2.10E+09	6.77E+07	1.23E+08	108.6	181.8	1.7	1.3

* Inhibition fold was calculated by normalizing the CFU recovery of antibiotic-treated samples to the untreated control at 6 hours.

ND indicates that no bacteria recovery after treatment and antibiotic susceptibility was not determined.

Supplemental Table 2. Strains used in this study.

Strain Name	Genotype	Background	Plasmid	Reference
TQ393	WT	*L. monocytogenes* 10403S		(1)
TQ290	WT::*pdeA*	*L. monocytogenes* 10403S	pPL2-*pdeA*	This work
TQ268	Δ*thyA*	*L. monocytogenes* 10403S		This work
TQ288	Δ*thyA*::*pdeA*	*L. monocytogenes* 10403S	pPL2-*pdeA*	This work
TQ322	Δ*thyA*::$pdeA_{84\text{-}657}$	*L. monocytogenes* 10403S	pPL2-$pdeA_{84\text{-}657}$	This work
TQ326	Δ*thyA*::$pdeA_{\text{DHH-AAA}}$	*L. monocytogenes* 10403S	pPL2-$pdeA_{\text{DHH-AAA}}$	This work
TQ304	Δ*thyA*Δ*pstA*	*L. monocytogenes* 10403S		This work
TQ306	Δ*thyA*Δ*pstA*::*pdeA*	*L. monocytogenes* 10403S	pPL2-*pdeA*	This work
TQ452	Δ*dacA*::*disA*	*L. monocytogenes* 10403S	pLIV2-*disA*	(2)
TQ446	Δ*dacA*::*dacA*	*L. monocytogenes* 10403S	pLIV2-*dacA*	(2)
TQ449	Δ*dacA*::*dacA* Δ*pstA*	*L. monocytogenes* 10403S	pLIV2-*dacA*	This work
TQ282	Δ*dacA*::*dacA* Δ*thyA*	*L. monocytogenes* 10403S	pLIV2-*dacA*	This work
TQ305	Δ*dacA*::*dacA* Δ*thyA*Δ*pstA*	*L. monocytogenes* 10403S	pLIV2-*dacA*	This work
TQ444	SM10	*E. coli* SM10		
TQ443	XL1B	*E. coli* XL1Blue		

Supplemental Table 3. Oligonucleotides used in this study

Oligo ID	Oligo Name	Sequence (5' to 3')	Description	Reference
		**Making *thyA* knockout mutants**		
189	*thyA*_U1	GGGGTACCTAATTTCTTTCATACGTATAAAAGTGTAATTG*		This work
190	*thyA*_U2	CAATACGCGGAATAAGGAGTGCGAACGATGTAAGGGAGGTTTTCTTGGATGATTATTTTT		This work
191	*thyA*_D1	AAAAATAATCATCCAAGAAAACCTCCCTTACATCGTTCGCACTCCTTATTCCGCGTATTG		This work
192	*thyA*_D2	AGTCTAGAACGACTTAGATACGCAAACATTAACCGTTTTAGA		This work
		**Overexpression of PdeA and its mutants using the pPL2 vector**		
199	*pdeA*_F	ACGCGTCGACATGTCAGGCTATTTTCAAAAACGAA	PdeA	This work
200	*pdeA*_R	ATTTGCGGCCGCTTATGTTTCTCCCTTCCAATACG	PdeA	This work
218	PdeA$_{84-657}$_F	ACGCGTCGACATGCCGATGGGAATACTGCTGTA	PdeA$_{84-657}$	This work
219	PdeA$_{DHH}$_R	TCCCAACAAATTCCTCTGAACGACGGgcAgcAgCGACAACAACCACATTCGTAGCTGA	PdeA$_{DHH-AAA}$	This work
220	PdeA$_{DHH}$_F	TCAGCTACGAATGTGGTTGTTGTCGcTgcTgcCCGTCGTTCAGAGGAATTTGTTGGGA	PdeA$_{DHH-AAA}$	This work
		**Complementation of *pstA* and its mutants using the pBAV1K-E vector**		
232	P_*pstA*_F	CTAGTCTAGAATATGGAACAAGTTTTACAAGTG	PstA	This work
233	P_*pstA*_R	CTAGACTAGTCTAAAAATGATGGAAACTCTCAAC		This work
		**Confirmation of *Lm* knockout mutants**		
195	*thyA*_KO_F	GCAAGCAGGCAACGTTTCTAC	Δ*thyA*	This work
196	*thyA*_KO_R	TAATGTATTACTACTAGATGAGCCGACC	Δ*thyA*	This work
241	*pstA*_KO_F	TAAAGCTTGGAACAAGTTAACAAGCAA	Δ*pstA*	This work
242	*PstA*_KO_R	TCGCTTGGATAAAGAAGATATTACCTAC	Δ*pstA*	This work
		**Plasmid sequencing primers**		
141	PBAV_1	ACGCTCTATCCCAACTGGCTCAAG	pBAV1K-E	This work
142	PBAV_2	CTTGAGCCAGTTGGGATAGAGCGT	pBAV1K-E	This work
351	pPL2_mcs_F	AGCTGTGACCGTCTCCG	pPL2	This work
352	pPL2_mcs_R	GACAACTTCTTCGCCCCCG	pPL2	This work
353	PliM_mcs_F	GTGTGGAATTGTGAGCGGATAAC	pLiM	This work
354	PliM_mcs_R	GGCGATTAAGTTGGGTAACGC	pLiM	This work

* Restriction enzyme recognition sites are highlighted in red

References

1. Becavin C, Bouchier C, Lechat P, Archambaud C, Creno S, Gouin E, Wu Z, Kuhbacher A, Brisse S, Pucciarelli MG, Garcia-del Portillo F, Hain T, Portnoy DA, Chakraborty T, Lecuit M, Pizarro-Cerda J, Moszer I, Bierne H, Cossart P. 2014. Comparison of widely used *Listeria monocytogenes* strains EGD, 10403S, and EGD-e highlights genomic variations underlying differences in pathogenicity. mBio 5:e00969-14.
2. Witte CE, Whiteley AT, Burke TP, Sauer JD, Portnoy DA, Woodward JJ. 2013. Cyclic di-AMP is critical for *Listeria monocytogenes* growth, cell wall homeostasis, and establishment of infection. mBio 4:e00282-13.
